# Adverse outcomes in chronic myeloid leukemia patients treated with tyrosine kinase inhibitors: Follow‐up of patients diagnosed 2002–2017 in a complete coverage and nationwide agnostic register study

**DOI:** 10.1002/ajh.26463

**Published:** 2022-01-20

**Authors:** Torsten Dahlén, Gustaf Edgren, Per Ljungman, Hjalmar Flygt, Johan Richter, Ulla Olsson‐Strömberg, Hans Wadenvik, Arta Dreimane, Kristina Myhr‐Eriksson, Jingcheng Zhao, Anders Själander, Martin Höglund, Leif Stenke

**Affiliations:** ^1^ Department of Medicine Solna, Clinical Epidemiology Division Karolinska Institutet Stockholm Sweden; ^2^ Department of Hematology Karolinska University Hospital Stockholm Sweden; ^3^ Department of Cardiology Södersjukhuset Stockholm Sweden; ^4^ Department of Cellular Therapy and Allogeneic Stem Cell Transplantation Karolinska University Hospital Huddinge, Stockholm Sweden; ^5^ Department of Medicine Solna Karolinska Institutet Stockholm Sweden; ^6^ Department of Medical Science and Division of Hematology University Hospital Uppsala Sweden; ^7^ Department of Hematology, Oncology and Radiation Physics Skåne University Hospital Lund Sweden; ^8^ Section of Hematology Sahlgrenska University Hospital Gothenburg Sweden; ^9^ Department of Hematology Linköping University Hospital Linköping Sweden; ^10^ Department of Hematology Sunderby Hospital Luleå Sweden; ^11^ Department of Public Health and Clinical Medicine Umeå University Sundsvall Sweden

## Abstract

Tyrosine kinase inhibitors (TKIs) have profoundly improved the clinical outcome for patients with chronic myeloid leukemia (CML), but their overall survival is still subnormal and the treatment is associated with adverse events. In a large cohort‐study, we assessed the morbidity in 1328 Swedish CML chronic phase patients diagnosed 2002–2017 and treated with TKIs, as compared to that in carefully matched control individuals. Several Swedish patient registers with near‐complete nationwide coverage were utilized for data acquisition. Median follow‐up was 6 (IQR, 3–10) years with a total follow‐up of 8510 person‐years for the full cohort. Among 670 analyzed disease categories, the patient cohort showed a significantly increased risk in 142 while, strikingly, no category was more common in controls. Increased incidence rate ratios/IRR (95% CI) for more severe events among patients included acute myocardial infarction (AMI) 2.0 (1.5–2.6), heart failure 2.6 (2.2–3.2), pneumonia 2.8 (2.3–3.5), and unspecified sepsis 3.5 (2.6–4.7). When comparing patients on 2nd generation TKIs vs. imatinib in a within‐cohort analysis, nilotinib generated elevated IRRs for AMI (2.9; 1.5–5.6) and chronic ischemic heart disease (2.2; 1.2–3.9), dasatinib for pleural effusion (11.6; 7.6–17.7) and infectious complications, for example, acute upper respiratory infections (3.0; 1.4–6.0). Our extensive real‐world data reveal significant risk increases of severe morbidity in TKI‐treated CML patients, as compared to matched controls, particularly for 2nd generation TKIs. Whether this increased morbidity may also translate into increased mortality, thus preventing CML patients to achieve a normalized overall survival, needs to be further explored.

## 1 INTRODUCTION

Chronic phase (CP) chronic myeloid leukemia (CML) is characterized by the fusion gene BCR‐ABL1, coding for an oncoprotein that acts as a tyrosine kinase.^1^ With the clinical introduction of imatinib, and subsequently other targeted tyrosine kinase inhibitors (TKIs), during the recent 20 years, the prognosis for CML patients has improved dramatically. The 5‐year relative survival rates for CML patients younger than 70 years of age are now approaching those of the general population.^2^, ^3^, ^4^ Compared to imatinib, recommended doses of the newer 2nd‐ and 3rd‐generation TKIs (dasatinib, nilotinib, bosutinib, and ponatinib) generally induce more rapid and deeper molecular responses, as assessed by quantitative RT‐PCR of the fusion‐transcript.^5^, ^6^, ^7^, ^8^, ^9^ However, this has not translated into any clear differences between the various TKIs regarding overall survival.^10^ Most TKIs are considered safe and well‐tolerated, based mainly on short‐term tolerability data from randomized trials.^11^ Dose‐limiting toxicities typically reach only grade 2 on the Common Terminology Criteria for Adverse Events (CTC‐AE) scale. Since some of the TKIs have been available for less than 10 years and the number of patients diagnosed with CML is comparatively small, systematic studies of long term‐toxicity have been scarce. We have previously used data from the Swedish CML Register to study TKI‐related cardiovascular toxicity.^12^ The issue was highlighted by reports on an increased incidence of mainly arterial events.^13^, ^14^ It has been suggested that this type of toxicity may help explain why the new generation of TKIs have not demonstrated superiority over imatinib in terms of overall survival. In our previous publication, we were limited to patients diagnosed between 2002 and 2012 with relatively limited duration of follow‐up in the patients treated with the newer TKIs nilotinib and dasatinib, and a very scarce use of bosutinib and ponatinib. There is a persistent paucity of data on the full range of morbidity in CML patients, which limits the possibility of tailoring TKI therapy to individual patients both with regards to short‐term response and to toxicity. With this background, we set out to explore the range of possible adverse events in a large group of CML patients treated with TKIs in a real‐world setting during extended time periods and to elaborate upon relations of these events to specific TKIs.

## 2 METHODS

### 2.1 Data sources and study population

We extracted data on all patients diagnosed with CP CML between January 1st, 2002 until December 31st, 2017 that were recorded in the nationwide Swedish CML Register. The Swedish CML Register is a quality of care register consisting of manually entered data on drug treatment and clinical and molecular disease characteristics from time of diagnosis and during follow‐up at 1, 2, 5, 10, and 15 years. Previous validations of the Swedish CML Register demonstrated more than 98% coverage of patients.^4^ Patients younger than 18 years were excluded as well as the few patients who were treated strictly with a palliative treatment intent (e.g. hydroxyurea without subsequent addition of TKI).

From the Swedish Total Population Register, a control population was selected with 5 random controls per CML patient, matched for year of birth, sex, and place of residence at the time of diagnosis of the index patient. Using unique personal identity numbers assigned to all Swedish citizens, we then extracted information concerning all non‐CML‐specific health care from the nationwide Swedish Patient and Cause of Death Registers.^15^, ^16^, ^17^ All Swedish healthcare providers are required to contribute data to the Patient Register, which thus covers all inpatient care in Sweden from 1987 and all specialist outpatient care since 2001. The Patient Register has been validated for a large set of outcomes.^16^ All diagnoses and causes of death were coded using the 10th revision of the international classification of disease (ICD‐10) throughout the study period.

Data on current TKI treatment was ascertained using a two‐pronged approach. Since 2005, all dispensed prescriptions were available through linkage with the nationwide Prescribed Drug Register.^18^ Earlier data, from 2002 to 2005, were instead extracted from the CML Register, where treatment is recorded both at diagnosis and during follow‐up. Of note, treatment data from the CML Register were also available for patients enrolled in clinical trials where drugs were supplied by the sponsor and thus not recorded in the Prescribed Drug Register. In subjects receiving multiple TKIs, follow‐up for each drug started at the date of the first dispensation of each treatment and continued until the day before the end‐date of the next treatment or at the end of follow‐up, whichever occurs first.

### 2.2 Outcomes

Using data from the Patient and Cause of Death registers, we extracted information for all patients on events requiring care at a hospital or events registered as the cause of death, coded as International Classification of Disease (ICD) revision 10, and categorized these into 670 disease categories, excluding malignancies, hematological diseases, external causes of morbidity and mortality, as well as symptom‐based ICD‐codes. Details of how the disease categories were categorized are available in Table S1.

### 2.3 Study design and statistical analyses

In the first analysis, we compared the incidence of all disease categories to the matched control population. Here, patients were followed from the date of CML diagnosis until the date of first incident event in each disease category investigated, emigration, death, progression to accelerated phase or blast crisis, or until the last day of follow‐up (December 31st, 2018), whichever occurred first. To remove effects of disease progression not captured by specified progression date, we also terminated follow‐up 5 months before allogeneic hematopoietic stem cell transplantation. Similarly, the control population was followed from date of diagnosis of matched CML patient, until the first incident event in each disease category, emigration, death, or December 2018, whichever occurred first.

After excluding disease categories with no events, incidence rate ratios (IRRs), comparing the CML patients to matched controls, were computed for each disease category using Poisson regression models. All analyses were adjusted for sex, calendar period of observation, and attained age, the latter two by fitting restricted cubic spline functions with 3 knots placed according to Harrel's method.^19^

For disease categories where Lagrange multiplier test indicated over‐ or under‐dispersion using regular Poisson regression, we performed Poisson regression with empirical variance estimation to construct standard errors and *p* values. The false‐discovery rate (FDR), according to Benjamini and Hochberg, was applied to the first analysis to account for multiple comparisons and type I errors.^20^ False‐discovery rate was used in preference to Bonferroni correction to produce less conservative non‐family‐wise adjustments not to fail to detect disease categorizes with possible increased risks. Raw *p*‐values were presented with unadjusted 95% confidence intervals, adjusted *p*‐values are presented in Table S2.

In the second analysis, we proceeded to test for associations between individual TKIs and the risk of each disease category among all disease categories significantly more common in CML patients than among controls identified in the first analysis. In this analysis, we performed an internal comparison only within the CML population, adding Sokal risk category at diagnosis to the Poisson model. In addition, the models also included current TKI treatment as a time‐dependent covariate, allowing the variable to change as patient received different treatments. Modeling was otherwise conducted in a similar manner as in the first analysis using a two‐pronged approach. However, if over‐dispersion was detected in the Lagrange multiplier test, then the specific disease category was modeled in a quasi‐Poisson framework in order to handle the time‐dependent drug covariate and to specifically address over‐dispersions and deflate the type 1 error rate. In the second analysis, for TKIs with no events during follow‐up in a specific disease category were excluded. Current TKI treatment was treated as a time‐varying covariate by allowing TKI treatment to change in the model as the patient received different treatments, using the publicly available Stratify macro.^21^ In recognition of it being a strictly explorative and due to likely insufficient power, the main comparisons were based on raw *p* values are presented. However, results are also presented using Bonferroni adjustment.

To limit effects of health outcomes seen primarily in the initial untreated and initiation‐phase of TKI therapy in CML, and effects of the likely increased surveillance of patients at the time of their diagnosis, where they typically undergo frequent blood testing and diagnostic workups, we also performed a sensitivity analysis where start of follow‐up was delayed until 6 months after CML diagnosis. As this analysis included the same hypothesis test and covariates from the main analysis, but only investigating the sub‐population of FDR significant disease categories, Bonferroni‐adjustment was conducted to handle sub‐population adjustment for multiple testing not accounted for using FDR.

All statistical computations were conducted using SAS (SAS Institute v 9.4, California, USA).

### 2.4 Ethical considerations

This study has been approved by the Swedish Ethical Review Authority (ref. nr: 2020–05425).

## 3 RESULTS

We included 1328 patients with CP CML diagnosed between 2002 and 2017. The median follow‐up was 6 years (interquartile range [IQR], 3–10 years), and 329 patients accrued more than 10 years of follow‐up. A total of 613 (46%) of patients were female. Baseline characteristics of the CML and control cohorts are shown in Table 1. As is shown in Table 2, 1213 (91%), 379 (29%), 388 (29%), 52 (4%), and 25 (2%) patients ever received imatinib, dasatinib, nilotinib, bosutinib and ponatinib, respectively. Patients treated with ponatinib were younger (median age, 51 years) and in later lines of treatment, with 64% receiving ponatinib as a 3rd line or later TKI treatment (Table 2). Follow‐up for the full cohort was 8510 person‐years, with 5985, 1143, 1288, 61 and 21 person‐years for imatinib, dasatinib, nilotinib, bosutinib and ponatinib, respectively.

**TABLE 1:** Baseline characteristics for CML and control cohort, respectively

	CML cohort	Control cohort
*N*	1328	6640
Females, *N* (%)	613 (46)	3065 (46)
Age at diagnosis of CML patient, *N* (%)		
<40 years	206 (16)	1013 (15)
40–65 years	610 (46)	3140 (47)
>65 years	512 (39)	2487 (37)
Median age, years (IQR)	60 (46–71)	60 (46–71)
Year of diagnosis of CML patient, *N* (%)		
2002–2004	231 (17)	1155 (17)
2005–2007	221 (17)	1105 (17)
2008–2010	222 (17)	1110 (17)
2011–2013	283 (21)	1415 (21)
2014–2016	287 (22)	1435 (22)
2017	84 (6)	420 (6)
Duration of follow‐up, *N* (%)		
0–5 years	584 (44)	2357 (35)
5–10 years	415 (31)	2188 (33)
10–15 years	261 (20)	1605 (24)
Median (IQR)	6 (3–10)	7 (4–11)
>15 years	68 (5)	490 (7)

*Note*: Maximum follow‐up in the CML cohort was 17 years.

Abbreviation: IQR, interquartile range.

**TABLE 2:** Baseline characteristics of CML cohort

	Imatinib	Dasatinib	Nilotinib	Bosutinib	Ponatinib
*N*	1213	379	388	52	25
Female, *N* (%)	45	47	46	56	48
Age, median (IQR)	60 (46–70)	60 (45–69)	57 (45–69)	60 (45–64)	51 (44–62)
Year of diagnosis					
2002–2004	171	34	29	4	1
2005–2007	227	67	48	4	1
2008–2010	233	74	80	5	
2011–2013	237	82	136	21	15
2014–2016	264	95	80	16	8
2017	81	27	15	2	
Line of TKI treatment, *N* (%)					
First	1100 (91)	24 (6)	126 (32)	1 (2)	7 (28)
Second	104 (9)	290 (77)	163 (42)	5 (10)	2 (8)
Third	8 (1)	60 (16)	90 (23)	25 (48)	8 (32)
Fourth	0 (0)	5 (1)	8 (2)	21 (40)	7 (28)
Fifth	1 (0)	0 (0)	1 (0)	0 (0)	1 (4)
Follow‐up on treatment, years					
Median (IQR)	3.2 (1.1–7.8)	1.9 (0.8–4.7)	2.8 (0.8–5.6)	0.6 (0.2–1.6)	0.5 (0.2–1‐0)
Total	5990	1140	1290	60	20

Abbreviation: IQR, interquartile range.

**FIGURE 1:**
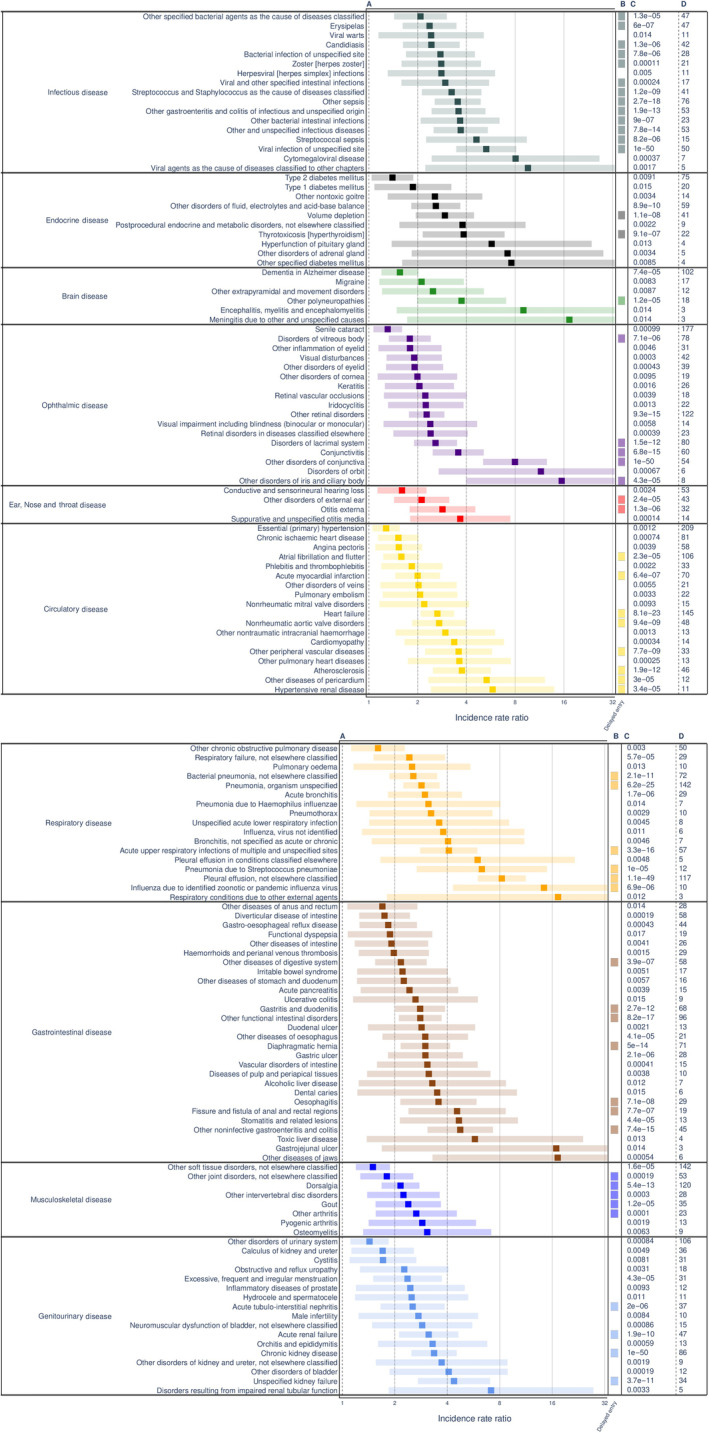
(A) demonstrates incidence rate ratios (IRRs) for significant finding after FDR adjustment for the CML population as compared to the control population: strong color demonstrating the point estimate and lighter color demonstrating 95% confidence intervals. (B) demonstrates the same analysis but findings significant after Bonferroni‐adjustment in a delayed entry model with 6 months from diagnosis of the significant findings from the first analysis. (C,D) column present raw *p* values for the main analysis and events, respectively [Color figure can be viewed at wileyonlinelibrary.com]

Out of 670 disease categories, a total of 405 disease categories remained for analysis in the comparison between CML patients and the control population after excluding 265 disease categories with too few events. Before adjusting for multiple testing in these disease categories, 169 categories were identified with significantly increased IRR as compared to the control population and no disease category demonstrated a decreased incidence. After FDR adjustment, 142 (84%) of these associations remained statistically significant (Figure 1 and Table S2 and Figure S1). Disease categories with increased risks were observed particularly among diseases of the circulatory, respiratory, ophthalmic, infectious, gastrointestinal, and genitourinary systems. Among disease categories with strong effects, we observed pleural effusion (IRR, 8.2; 95% CI, 6.2–10.8), pneumonia with unspecified organism (IRR, 2.8; 95% CI, 2.3–3.5), heart failure (IRR, 2.6; 95% CI, 2.2–3.2), other sepsis (IRR, 3.5; 95% CI, 2.6–4.7), other functional intestinal disorders (IRR, 2.8; 95% CI, 2.2–3.5), acute upper respiratory infections (IRR, 4.1; 95% CI, 2.9–5.7), conjunctivitis (IRR, 3.6; 95% CI, 2.6–4.9), and other noninfective gastroenteritis and colitis (IRR, 4.7; 95% CI, 3.2–7.0). In the delayed entry model, where follow‐up was started 6 months after diagnosis, 54 (38%) associations were still statistically significant after Bonferroni adjustment, as compared to the control population. Of note, the risk of acute myocardial infarction remained elevated with similar risk estimates in the delayed entry model, with IRRs of 2.0 (95% CI, 1.5–2.6) and 2.1 (95% CI, 1.5–2.7) in the main and delayed‐entry model, respectively. Risk of retinal vascular disorders was only statistically significantly elevated when including the full follow‐up and not in the delayed entry model, with IRRs of 2.2 (95% CI, 1.3–3.8) and 1.7 (95% CI, 0.9–3.0), respectively. Most infectious diagnoses (i.e., in the first chapter of the ICD system), remained statistically significant also in the delayed entry model.

When considering the effects of specific TKIs, investigating the significant disease categories from the first analysis, we demonstrated an association between individual TKIs and disease categories in 41 (29%) of the 139 investigated disease categories as compared to imatinib (Table 3 and Figure S1). For nilotinib, the risk was statistically significantly elevated for several cardiovascular outcomes, most notably acute myocardial infarction (IRR, 2.9; 95% CI, 1.5–5.6) and chronic ischemic heart disease (IRR, 2.2; 95% CI, 1.2–3.9). For dasatinib, statistically significantly increased risks were observed for pleural effusion (IRR, 11.6; 95% CI,7.6–17.7) and infectious complications, e.g. erysipelas (IRR, 3.7; 95% CI, 1.4–9.7) and acute upper respiratory infections (IRR, 3.0; 95% CI, 1.4–6.0). On account of the small number of patients treated with bosutinib or ponatinib and short follow‐up, any associations observed for these drugs were coupled with large standard errors.

**TABLE 3:** Significant findings from within cohort analysis, TKI specific, as compared to imatinib as reference

	Events	Incidence rate ratio (95% CI)	Raw *p* value	Bonferroni robust
Dasatinib				
Heart failure	28	2.2 (1.4–3.4)	.0005	
Gastric ulcer	6	3 (1.1–8.1)	.0349	
Erysipelas	16	3.7 (1.4–9.7)	.0082	
Other bacterial intestinal infections	6	3.5 (1.2–10.1)	.021	
Other gastroenteritis and colitis of infectious and unspecified origin	15	2.7 (1.4–5.1)	.0027	
Disorders of lacrimal system	3	0.3 (0.1–0.9)	.0345	
Pleural effusion, not elsewhere classified	66	11.6 (7.6–17.7)	<.0001	Yes
Acute upper respiratory infections of multiple and unspecified sites	12	3 (1.4–6.0)	.003	
Pneumothorax	5	10.6 (1.6–69.9)	.01	
Nilotinib				
Atherosclerosis	17	4.7 (2.3–9.5)	<.0001	Yes
Other peripheral vascular diseases	11	3.4 (1.5–7.7)	.0027	
Acute myocardial infarction	15	2.9 (1.5–5.6)	.0012	
Chronic ischaemic heart disease	17	2.2 (1.2–3.9)	.009	
Atrial fibrillation and flutter	23	1.8 (1.1–3.0)	.0171	
Essential (primary) hypertension	32	1.5 (1.0–2.3)	.0371	
Type 2 diabetes mellitus	15	2.2 (1.2–4.1)	.0142	
Acute tubulo‐interstitial nephritis	9	3 (1.2–7.0)	.0147	
Cystitis	7	2.6 (1.0–6.8)	.0474	
Other disorders of urinary system	20	1.8 (1.0–3.0)	.0384	
Visual disturbances	14	3.8 (1.8–7.8)	.0003	
Pleural effusion, not elsewhere classified	13	2 (1.0–3.9)	.0352	
Bosutinib				
Heart failure	2	5.3 (1.3–22.0)	.0229	
Unspecified kidney failure	2	7 (1.5–31.5)	.0118	
Gastritis and duodenitis	2	5.6 (1.3–24.6)	.0216	
Other bacterial intestinal infections	1	16.9 (1.9–152.0)	.0115	
Ponatinib				
Angina pectoris	1	15.6 (2.0–119.0)	.0081	
Atherosclerosis	1	13.7 (1.7–108.4)	.0131	
Other peripheral vascular diseases	1	12.2 (1.5–98.0)	.0189	
Chronic ischaemic heart disease	1	8.7 (1.2–64.8)	.0351	
Atrial fibrillation and flutter	1	7.6 (1.0–55.6)	.0462	
Essential (primary) hypertension	2	7.3 (1.8–30.0)	.0062	
Other disorders of fluid, electrolyte and acid–base balance	2	16.4 (3.8–71.0)	.0002	
Type 2 diabetes mellitus	1	7.8 (1.0–58.7)	.0459	
Cystitis	1	24.4 (2.9–202.4)	.0031	
Excessive, frequent and irregular menstruation	1	11 (1.3–94.4)	.0289	
Other diseases of esophagus	1	23.2 (1.8–297.2)	.0157	
Oesophagitis	1	22.3 (1.6–312.9)	.0211	
Diverticular disease of intestine	1	10.7 (1.4–80.2)	.0215	
Zoster [herpes zoster]	1	24.2 (2.9–203.0)	.0034	
Candidiasis	2	23.3 (5.1–106.5)	<.0001	Yes
Keratitis	1	14.2 (1.5–133.3)	.0202	
Acute upper respiratory infections of multiple and unspecified sites	1	19.6 (2.5–154.4)	.0046	

## 4 DISCUSSION

In this nationwide cohort study, including almost all CP CML patients diagnosed in Sweden between 2002 and 2017, we describe the full spectrum of morbidity associated with CML and TKI treatment. After exclusions, we studied the occurrence of 405 disease categories, both comparing CML patients to the general population and patients treated with different TKIs. The cumulative data set is extensive and complex, but some observations may deserve particular attention. First, for 169 categories CML patients were found carrying a significantly increased risk as compared to matched controls, while the reverse could not be observed for any category. This clearly indicates an inferior health status, and possibly an inferior quality of life, among CML patients. Among the most notable, not exceedingly rare events (n > 50) with at least a significantly doubling of IRRs after Bonferroni adjustment were several potentially severe conditions, such as “other sepsis,” pneumonia, pleural effusion, acute myocardial infarction, heart failure, and gastritis/duodenitis. Whether this also translated into lethal complications among affected individuals, thus counteracting the clear benefits of TKI treatment, is not known. These adverse events have all been attributed to TKI treatment in earlier publications,^22^ but our data expands and deepens the elucidation of their prevalence. Comparing the outcome of patients treated with imatinib versus those treated with later generation TKIs provided further insights. The risk of acute myocardial infarction was found to be increased 3‐fold in the nilotinib‐treated group, while the risks of several infectious diseases were particularly elevated for dasatinib‐treated patients. This may have important clinical implications. CML patients during TKI therapy have not generally been considered to be in a clearly immunosuppressed state, an assumption supported by recent data on how these patients are affected by covid‐19.^23^ Our data indicate that the impact of prolonged TKI exposure might be more detrimental than previously thought. Extended studies appear warranted. Regarding less severe AE, it is also of note that the risks of a range of ophthalmic disease were elevated, both comparing the CML patients to the general population, as well as comparing CML patients treated with imatinib to those on 2nd generation TKI regimens. It is true that the adverse events listed in this report have been described in earlier publications,^22^, ^24^, ^25^ but our data provide expanded and deeper insights, supplying carefully matched control populations with extended follow‐up.

Besides including a full nationwide cohort, this study has several strengths, most notably the long follow‐up and the high‐resolution health‐care data. The detailed data on individual TKI therapy and the complete and detailed outcomes ascertainment from the national Patient Register, ensures a high reliability of both exposure and outcome data. In addition, the methodological approach should effectively bypass *researcher bias* by agnostically, without any pre‐conceived ideas concerning the relation of an exposure and outcome, investigating associations. As CML serves as a founding disease for modern precision‐based cancer therapy – where therapeutics effectively target a specific cancerous process – we need to be alert and develop robust surveillance methods to ensure the safety of all new targeted therapies, allowing the capture of a broad range of adverse events, and in an exploratory setting follow‐up and identify possible off‐target effects. One such method is by including the full population of patients in high quality registers allowing for complete follow‐up and applying methods such as these to the data.

However, with the agnostic approach comes also statistical limitations, where it is necessary to strike a suitable balance between an inflated type 1 error rate (i.e., with large numbers of false positive findings) and a limited statistical power. In the main analysis, we therefore implemented the commonly employed FDR method to handle multiple testing, widely used in genetic association studies. An alternative method would have been to use a family‐wide method, reducing the type 1 error rate to a minimum, but at substantial cost of limiting statistical power. Given the exploratory nature of the study and that the aim was to characterize the full spectrum of morbidity, to generate data for future confirmatory studies in other CML populations, selecting FDR as adjustment method was a natural choice.

An additional limitation comes from the structure of the investigated disease categories, which was based on 3‐digit ICD‐10 codes and which may therefore lead to some outcome heterogeneity as such disease categories sometimes include more than one distinct disease, with different etiology. While none of the statistically significant associations that we detected were related to heterogenous outcome categories, it is still possible that we failed to detect potentially relevant associations with diseases that were classified together with other diseases in heterogenous outcome categories. Non‐acute and less severe conditions are also more likely to be discovered in CML patients due to closer monitoring than in the general population. Another limitation is the limited number – and short follow‐up – of patients treated with ponatinib and bosutinib, which may ostensibly depict these drugs as being safer because of limited data. Furthermore, a general effect of using health‐visit data to study disease occurrence among patients with one chronic condition is how associations with other diseases may be driven by increased detection, either due to improved surveillance from repeated health care contacts, or from patients being more prone to seek healthcare for mild symptoms because of the underlying illness. Such effects should mainly impact milder, non‐acute diseases and could be seen as an example of reversed causality. However, it is generally complex to disentangle such incidental findings from true effects of the underlying disease or treatment. To counter these findings, possibly identified as a consequence of surveillance in newly diagnosed CML, we additionally used a delayed entry model, still demonstrating a multitude of disease categories with increased risks even after conservative Bonferroni adjustment.

In conclusion, data from this large “real‐world” cohort study with close to full nationwide coverage extensively and specifically identify and pinpoint a number of severe adverse events linked to CML patients subjected to prolonged treatment with TKI (in particular with 2nd generation drugs), as compared to outcomes for carefully matched controls. The full clinical implications of these findings are still somewhat unclear, but they may well provide important novel insights into the long‐term morbidity and mortality of CML patients. In general, we believe that explorative studies using this or similar methodologies, capturing a large set of event types in large patient cohorts, constitute a fruitful approach to identify and quantify clinical risks associated with novel therapeutics, perhaps in particular off‐target effects by newly introduced precision‐based treatment regimens.

## CONFLICT OF INTEREST

Dr Dahlén reports funding outside the scope of this work from Novartis. Dr Edgren reports funding from outside this work from Celgene.

## AUTHOR CONTRIBUTIONS

Torsten Dahlén had full access to all the data in the study and takes responsibility for the integrity of the data and the accuracy of the data analysis. *Concept and design*: Torsten Dahlén, Gustaf Edgren, Leif Stenke, Martin Höglund. *Acquisition*, *analysis*, *or interpretation of data*: Torsten Dahlén, Gustaf Edgren, Leif Stenke, Martin Höglund, Anders Själander. *Drafting of the manuscript*: Torsten Dahlén. *Critical revision of the manuscript for important intellectual content*: All authors. *Statistical analysis*: Torsten Dahlén. *Obtained funding*: Martin Höglund. *Administrative*, *technical*, *or material support*: Torsten Dahlén. *Supervision*: Torsten Dahlén, Gustaf Edgren, Leif Stenke.

## PATIENT CONSENT

The current study is supported by the general consent given for national quality‐of‐care registers in Sweden, where patients are informed of participation in quality‐of‐care registers and the possibility to actively withdraw from such register.

## Supporting information


**Appendix** **S1** Supporting Information

## Data Availability

Due to laws guarding the integrity of the Swedish citizens, no patient‐level data can be made available. Upon request, aggregate data with different levels of resolution may be possible to provide.
