# Supplementary material for: Adverse outcomes in chronic myeloid leukemia patients treated with tyrosine kinase inhibitors: Follow‐up of patients diagnosed 2002–2017 in a complete coverage and nationwide agnostic register study
Source: Am J Hematol. 2022 Jan 20;97(4):421–30. doi: 10.1002/ajh.26463 (PMC9306877; doi:10.1002/ajh.26463)
Supplement: Supplementary file 1 — Appendix S1 Supporting Information [file AJH-97-421-s001.docx]

**Supplements**

**List of supplements**

Supplement 1 – Table 1. Disease categories investigated and corresponding ICD codes.

Supplement 2 – Table 2. Results from main analysis presented in tabular format.

Supplement 3 – Table 3. Results from delayed-entry analysis presented in tabular format.

Supplements 4 – Figure summarizing all findings.

**Supplement 1 – Table 1. Disease categories investigated and corresponding ICD codes.**

| **Main chapter number** | **Category** | **Main ICD Chapter** | **ICD codes** |
| --- | --- | --- | --- |
| 1 | Cholera | Certain infectious and parasitic diseases | A00 |
| 1 | Typhoid and paratyphoid fevers | Certain infectious and parasitic diseases | A01 |
| 1 | Other Salmonella infections | Certain infectious and parasitic diseases | A02 |
| 1 | Other bacterial intestinal infections | Certain infectious and parasitic diseases | A04 |
| 1 | Other bacterial foodborne intoxications, not elsewhere classified | Certain infectious and parasitic diseases | A05 |
| 1 | Amoebiasis | Certain infectious and parasitic diseases | A06 |
| 1 | Other protozoal intestinal diseases | Certain infectious and parasitic diseases | A07 |
| 1 | Viral and other specified intestinal infections | Certain infectious and parasitic diseases | A08 |
| 1 | Other gastroenteritis and colitis of infectious and unspecified origin | Certain infectious and parasitic diseases | A09 |
| 1 | Respiratory tuberculosis, bacteriologically and histologically confirmed | Certain infectious and parasitic diseases | A15 |
| 1 | Respiratory tuberculosis, not confirmed bacteriologically or histologically | Certain infectious and parasitic diseases | A16 |
| 1 | Tuberculosis of other organs | Certain infectious and parasitic diseases | A18 |
| 1 | Miliary tuberculosis | Certain infectious and parasitic diseases | A19 |
| 1 | Tularaemia | Certain infectious and parasitic diseases | A21 |
| 1 | Anthrax | Certain infectious and parasitic diseases | A22 |
| 1 | Erysipeloid | Certain infectious and parasitic diseases | A26 |
| 1 | Other zoonotic bacterial diseases, not elsewhere classified | Certain infectious and parasitic diseases | A28 |
| 1 | Infection due to other mycobacteria | Certain infectious and parasitic diseases | A31 |
| 1 | Listeriosis | Certain infectious and parasitic diseases | A32 |
| 1 | Diphtheria | Certain infectious and parasitic diseases | A36 |
| 1 | Scarlet fever | Certain infectious and parasitic diseases | A38 |
| 1 | Meningococcal infection | Certain infectious and parasitic diseases | A39 |
| 1 | Streptococcal sepsis | Certain infectious and parasitic diseases | A40 |
| 1 | Other sepsis | Certain infectious and parasitic diseases | A41 |
| 1 | Actinomycosis | Certain infectious and parasitic diseases | A42 |
| 1 | Bartonellosis | Certain infectious and parasitic diseases | A44 |
| 1 | Erysipelas | Certain infectious and parasitic diseases | A46 |
| 1 | Other bacterial diseases, not elsewhere classified | Certain infectious and parasitic diseases | A48 |
| 1 | Bacterial infection of unspecified site | Certain infectious and parasitic diseases | A49 |
| 1 | Congenital syphilis | Certain infectious and parasitic diseases | A50 |
| 1 | Late syphilis | Certain infectious and parasitic diseases | A52 |
| 1 | Other and unspecified syphilis | Certain infectious and parasitic diseases | A53 |
| 1 | Gonococcal infection | Certain infectious and parasitic diseases | A54 |
| 1 | Other sexually transmitted chlamydial diseases | Certain infectious and parasitic diseases | A56 |
| 1 | Chancroid | Certain infectious and parasitic diseases | A57 |
| 1 | Trichomoniasis | Certain infectious and parasitic diseases | A59 |
| 1 | Anogenital herpesviral [herpes simplex] infection | Certain infectious and parasitic diseases | A60 |
| 1 | Other predominantly sexually transmitted diseases, not elsewhere classified | Certain infectious and parasitic diseases | A63 |
| 1 | Unspecified sexually transmitted disease | Certain infectious and parasitic diseases | A64 |
| 1 | Yaws | Certain infectious and parasitic diseases | A66 |
| 1 | Other spirochaetal infections | Certain infectious and parasitic diseases | A69 |
| 1 | Chlamydia psittaci infection | Certain infectious and parasitic diseases | A70 |
| 1 | Trachoma | Certain infectious and parasitic diseases | A71 |
| 1 | Other diseases caused by chlamydiae | Certain infectious and parasitic diseases | A74 |
| 1 | Acute poliomyelitis | Certain infectious and parasitic diseases | A80 |
| 1 | Rabies | Certain infectious and parasitic diseases | A82 |
| 1 | Tick-borne viral encephalitis | Certain infectious and parasitic diseases | A84 |
| 1 | Other viral encephalitis, not elsewhere classified | Certain infectious and parasitic diseases | A85 |
| 1 | Unspecified viral encephalitis | Certain infectious and parasitic diseases | A86 |
| 1 | Viral meningitis | Certain infectious and parasitic diseases | A87 |
| 1 | Unspecified viral infection of central nervous system | Certain infectious and parasitic diseases | A89 |
| 1 | Other viral haemorrhagic fevers, not elsewhere classified | Certain infectious and parasitic diseases | A98 |
| 1 | Unspecified viral haemorrhagic fever | Certain infectious and parasitic diseases | A99 |
| 1 | Herpesviral [herpes simplex] infections | Certain infectious and parasitic diseases | B00 |
| 1 | Varicella [chickenpox] | Certain infectious and parasitic diseases | B01 |
| 1 | Zoster [herpes zoster] | Certain infectious and parasitic diseases | B02 |
| 1 | Smallpox | Certain infectious and parasitic diseases | B03 |
| 1 | Monkeypox | Certain infectious and parasitic diseases | B04 |
| 1 | Measles | Certain infectious and parasitic diseases | B05 |
| 1 | Viral warts | Certain infectious and parasitic diseases | B07 |
| 1 | Other viral infections characterized by skin and mucous membrane | Certain infectious and parasitic diseases | B08 |
| 1 | Unspecified viral infection characterized by skin and mucous | Certain infectious and parasitic diseases | B09 |
| 1 | Acute hepatitis A | Certain infectious and parasitic diseases | B15 |
| 1 | Acute hepatitis B | Certain infectious and parasitic diseases | B16 |
| 1 | Other acute viral hepatitis | Certain infectious and parasitic diseases | B17 |
| 1 | Chronic viral hepatitis | Certain infectious and parasitic diseases | B18 |
| 1 | Unspecified viral hepatitis | Certain infectious and parasitic diseases | B19 |
| 1 | Human immunodeficiency virus [HIV] disease resulting in infectious | Certain infectious and parasitic diseases | B20 |
| 1 | Human immunodeficiency virus [HIV] disease resulting in other conditions | Certain infectious and parasitic diseases | B23 |
| 1 | Unspecified human immunodeficiency virus [HIV] disease | Certain infectious and parasitic diseases | B24 |
| 1 | Cytomegaloviral disease | Certain infectious and parasitic diseases | B25 |
| 1 | Mumps | Certain infectious and parasitic diseases | B26 |
| 1 | Infectious mononucleosis | Certain infectious and parasitic diseases | B27 |
| 1 | Viral conjunctivitis | Certain infectious and parasitic diseases | B30 |
| 1 | Other viral diseases, not elsewhere classified | Certain infectious and parasitic diseases | B33 |
| 1 | Viral infection of unspecified site | Certain infectious and parasitic diseases | B34 |
| 1 | Dermatophytosis | Certain infectious and parasitic diseases | B35 |
| 1 | Other superficial mycoses | Certain infectious and parasitic diseases | B36 |
| 1 | Candidiasis | Certain infectious and parasitic diseases | B37 |
| 1 | Aspergillosis | Certain infectious and parasitic diseases | B44 |
| 1 | Zygomycosis | Certain infectious and parasitic diseases | B46 |
| 1 | Other mycoses, not elsewhere classified | Certain infectious and parasitic diseases | B48 |
| 1 | Unspecified mycosis | Certain infectious and parasitic diseases | B49 |
| 1 | Plasmodium vivax malaria | Certain infectious and parasitic diseases | B51 |
| 1 | Other parasitologically confirmed malaria | Certain infectious and parasitic diseases | B53 |
| 1 | Unspecified malaria | Certain infectious and parasitic diseases | B54 |
| 1 | Leishmaniasis | Certain infectious and parasitic diseases | B55 |
| 1 | Toxoplasmosis | Certain infectious and parasitic diseases | B58 |
| 1 | Pneumocystosis | Certain infectious and parasitic diseases | B59 |
| 1 | Other protozoal diseases, not elsewhere classified | Certain infectious and parasitic diseases | B60 |
| 1 | Unspecified protozoal disease | Certain infectious and parasitic diseases | B64 |
| 1 | Schistosomiasis [bilharziasis] | Certain infectious and parasitic diseases | B65 |
| 1 | Echinococcosis | Certain infectious and parasitic diseases | B67 |
| 1 | Taeniasis | Certain infectious and parasitic diseases | B68 |
| 1 | Hookworm diseases | Certain infectious and parasitic diseases | B76 |
| 1 | Strongyloidiasis | Certain infectious and parasitic diseases | B78 |
| 1 | Trichuriasis | Certain infectious and parasitic diseases | B79 |
| 1 | Enterobiasis | Certain infectious and parasitic diseases | B80 |
| 1 | Other intestinal helminthiases, not elsewhere classified | Certain infectious and parasitic diseases | B81 |
| 1 | Other helminthiases | Certain infectious and parasitic diseases | B83 |
| 1 | Pediculosis and phthiriasis | Certain infectious and parasitic diseases | B85 |
| 1 | Scabies | Certain infectious and parasitic diseases | B86 |
| 1 | Other infestations | Certain infectious and parasitic diseases | B88 |
| 1 | Unspecified parasitic disease | Certain infectious and parasitic diseases | B89 |
| 1 | Sequelae of tuberculosis | Certain infectious and parasitic diseases | B90 |
| 1 | Sequelae of poliomyelitis | Certain infectious and parasitic diseases | B91 |
| 1 | Sequelae of other and unspecified infectious and parasitic diseases | Certain infectious and parasitic diseases | B94 |
| 1 | Streptococcus and Staphylococcus as the cause of diseases classified | Certain infectious and parasitic diseases | B95 |
| 1 | Other specified bacterial agents as the cause of diseases classified | Certain infectious and parasitic diseases | B96 |
| 1 | Viral agents as the cause of diseases classified to other chapters | Certain infectious and parasitic diseases | B97 |
| 1 | Other specified infectious agents as the cause of diseases classified | Certain infectious and parasitic diseases | B98 |
| 1 | Other and unspecified infectious diseases | Certain infectious and parasitic diseases | B99 |
| 4 | Subclinical iodine-deficiency hypothyroidism | Endocrine, nutritional and metabolic dise | E02 |
| 4 | Other hypothyroidism | Endocrine, nutritional and metabolic dise | E03 |
| 4 | Other nontoxic goitre | Endocrine, nutritional and metabolic dise | E04 |
| 4 | Thyrotoxicosis [hyperthyroidism] | Endocrine, nutritional and metabolic dise | E05 |
| 4 | Thyroiditis | Endocrine, nutritional and metabolic dise | E06 |
| 4 | Other disorders of thyroid | Endocrine, nutritional and metabolic dise | E07 |
| 4 | Type 1 diabetes mellitus | Endocrine, nutritional and metabolic dise | E10 |
| 4 | Type 2 diabetes mellitus | Endocrine, nutritional and metabolic dise | E11 |
| 4 | Malnutrition-related diabetes mellitus | Endocrine, nutritional and metabolic dise | E12 |
| 4 | Other specified diabetes mellitus | Endocrine, nutritional and metabolic dise | E13 |
| 4 | Unspecified diabetes mellitus | Endocrine, nutritional and metabolic dise | E14 |
| 4 | Other disorders of pancreatic internal secretion | Endocrine, nutritional and metabolic dise | E16 |
| 4 | Hypoparathyroidism | Endocrine, nutritional and metabolic dise | E20 |
| 4 | Hyperparathyroidism and other disorders of parathyroid gland | Endocrine, nutritional and metabolic dise | E21 |
| 4 | Hyperfunction of pituitary gland | Endocrine, nutritional and metabolic dise | E22 |
| 4 | Hypofunction and other disorders of pituitary gland | Endocrine, nutritional and metabolic dise | E23 |
| 4 | Cushing syndrome | Endocrine, nutritional and metabolic dise | E24 |
| 4 | Adrenogenital disorders | Endocrine, nutritional and metabolic dise | E25 |
| 4 | Hyperaldosteronism | Endocrine, nutritional and metabolic dise | E26 |
| 4 | Other disorders of adrenal gland | Endocrine, nutritional and metabolic dise | E27 |
| 4 | Ovarian dysfunction | Endocrine, nutritional and metabolic dise | E28 |
| 4 | Testicular dysfunction | Endocrine, nutritional and metabolic dise | E29 |
| 4 | Disorders of puberty, not elsewhere classified | Endocrine, nutritional and metabolic dise | E30 |
| 4 | Polyglandular dysfunction | Endocrine, nutritional and metabolic dise | E31 |
| 4 | Diseases of thymus | Endocrine, nutritional and metabolic dise | E32 |
| 4 | Other endocrine disorders | Endocrine, nutritional and metabolic dise | E34 |
| 4 | Nutritional marasmus | Endocrine, nutritional and metabolic dise | E41 |
| 4 | Marasmic kwashiorkor | Endocrine, nutritional and metabolic dise | E42 |
| 4 | Unspecified severe protein-energy malnutrition | Endocrine, nutritional and metabolic dise | E43 |
| 4 | Protein-energy malnutrition of moderate and mild degree | Endocrine, nutritional and metabolic dise | E44 |
| 4 | Retarded development following protein-energy malnutrition | Endocrine, nutritional and metabolic dise | E45 |
| 4 | Unspecified protein-energy malnutrition | Endocrine, nutritional and metabolic dise | E46 |
| 4 | Vitamin A deficiency | Endocrine, nutritional and metabolic dise | E50 |
| 4 | Thiamine deficiency | Endocrine, nutritional and metabolic dise | E51 |
| 4 | Deficiency of other B group vitamins | Endocrine, nutritional and metabolic dise | E53 |
| 4 | Ascorbic acid deficiency | Endocrine, nutritional and metabolic dise | E54 |
| 4 | Vitamin D deficiency | Endocrine, nutritional and metabolic dise | E55 |
| 4 | Other vitamin deficiencies | Endocrine, nutritional and metabolic dise | E56 |
| 4 | Dietary zinc deficiency | Endocrine, nutritional and metabolic dise | E60 |
| 4 | Deficiency of other nutrient elements | Endocrine, nutritional and metabolic dise | E61 |
| 4 | Other nutritional deficiencies | Endocrine, nutritional and metabolic dise | E63 |
| 4 | Sequelae of malnutrition and other nutritional deficiencies | Endocrine, nutritional and metabolic dise | E64 |
| 4 | Localized adiposity | Endocrine, nutritional and metabolic dise | E65 |
| 4 | Obesity | Endocrine, nutritional and metabolic dise | E66 |
| 4 | Sequelae of hyperalimentation | Endocrine, nutritional and metabolic dise | E68 |
| 4 | Disorders of aromatic amino-acid metabolism | Endocrine, nutritional and metabolic dise | E70 |
| 4 | Other disorders of amino-acid metabolism | Endocrine, nutritional and metabolic dise | E72 |
| 4 | Lactose intolerance | Endocrine, nutritional and metabolic dise | E73 |
| 4 | Other disorders of carbohydrate metabolism | Endocrine, nutritional and metabolic dise | E74 |
| 4 | Disorders of sphingolipid metabolism and other lipid storage disorders | Endocrine, nutritional and metabolic dise | E75 |
| 4 | Disorders of glycosaminoglycan metabolism | Endocrine, nutritional and metabolic dise | E76 |
| 4 | Disorders of lipoprotein metabolism and other lipidaemias | Endocrine, nutritional and metabolic dise | E78 |
| 4 | Disorders of purine and pyrimidine metabolism | Endocrine, nutritional and metabolic dise | E79 |
| 4 | Disorders of porphyrin and bilirubin metabolism | Endocrine, nutritional and metabolic dise | E80 |
| 4 | Disorders of mineral metabolism | Endocrine, nutritional and metabolic dise | E83 |
| 4 | Cystic fibrosis | Endocrine, nutritional and metabolic dise | E84 |
| 4 | Amyloidosis | Endocrine, nutritional and metabolic dise | E85 |
| 4 | Volume depletion | Endocrine, nutritional and metabolic dise | E86 |
| 4 | Other disorders of fluid, electrolyte and acid–base balance | Endocrine, nutritional and metabolic dise | E87 |
| 4 | Other metabolic disorders | Endocrine, nutritional and metabolic dise | E88 |
| 4 | Postprocedural endocrine and metabolic disorders, not elsewhere classified | Endocrine, nutritional and metabolic dise | E89 |
| 4 | Nutritional and metabolic disorders in diseases classified elsewhere | Endocrine, nutritional and metabolic dise | E90 |
| 5 | Dementia in Alzheimer disease (G30.-†) | Mental and behavioural disorders | F00 |
| 5 | Vascular dementia | Mental and behavioural disorders | F01 |
| 5 | Dementia in other diseases classified elsewhere | Mental and behavioural disorders | F02 |
| 5 | Unspecified dementia | Mental and behavioural disorders | F03 |
| 5 | Delirium, not induced by alcohol and other psychoactive substances | Mental and behavioural disorders | F05 |
| 6 | Bacterial meningitis, not elsewhere classified | Diseases of the nervous system | G00 |
| 6 | Meningitis in bacterial diseases classified elsewhere | Diseases of the nervous system | G01 |
| 6 | Meningitis in other infectious and parasitic diseases classified elsewhere | Diseases of the nervous system | G02 |
| 6 | Meningitis due to other and unspecified causes | Diseases of the nervous system | G03 |
| 6 | Encephalitis, myelitis and encephalomyelitis | Diseases of the nervous system | G04 |
| 6 | Encephalitis, myelitis and encephalomyelitis in diseases classified | Diseases of the nervous system | G05 |
| 6 | Intracranial and intraspinal abscess and granuloma | Diseases of the nervous system | G06 |
| 6 | Sequelae of inflammatory diseases of central nervous system | Diseases of the nervous system | G09 |
| 6 | Huntington disease | Diseases of the nervous system | G10 |
| 6 | Hereditary ataxia | Diseases of the nervous system | G11 |
| 6 | Spinal muscular atrophy and related syndromes | Diseases of the nervous system | G12 |
| 6 | Systemic atrophies primarily affecting central nervous system in diseases | Diseases of the nervous system | G13 |
| 6 | Postpolio syndrome | Diseases of the nervous system | G14 |
| 6 | Parkinson disease | Diseases of the nervous system | G20 |
| 6 | Secondary parkinsonism | Diseases of the nervous system | G21 |
| 6 | Parkinsonism in diseases classified elsewhere | Diseases of the nervous system | G22 |
| 6 | Other degenerative diseases of basal ganglia | Diseases of the nervous system | G23 |
| 6 | Dystonia | Diseases of the nervous system | G24 |
| 6 | Other extrapyramidal and movement disorders | Diseases of the nervous system | G25 |
| 6 | Alzheimer disease | Diseases of the nervous system | G30 |
| 6 | Other degenerative diseases of nervous system, not elsewhere classified | Diseases of the nervous system | G31 |
| 6 | Other degenerative disorders of nervous system in diseases classified | Diseases of the nervous system | G32 |
| 6 | Multiple sclerosis | Diseases of the nervous system | G35 |
| 6 | Other acute disseminated demyelination | Diseases of the nervous system | G36 |
| 6 | Other demyelinating diseases of central nervous system | Diseases of the nervous system | G37 |
| 6 | Epilepsy | Diseases of the nervous system | G40 |
| 6 | Status epilepticus | Diseases of the nervous system | G41 |
| 6 | Migraine | Diseases of the nervous system | G43 |
| 6 | Other headache syndromes | Diseases of the nervous system | G44 |
| 6 | Transient cerebral ischaemic attacks and related syndromes | Diseases of the nervous system | G45 |
| 6 | Vascular syndromes of brain in cerebrovascular diseases (I60–I67†) | Diseases of the nervous system | G46 |
| 6 | Sleep disorders | Diseases of the nervous system | G47 |
| 6 | Disorders of trigeminal nerve | Diseases of the nervous system | G50 |
| 6 | Facial nerve disorders | Diseases of the nervous system | G51 |
| 6 | Disorders of other cranial nerves | Diseases of the nervous system | G52 |
| 6 | Cranial nerve disorders in diseases classified elsewhere | Diseases of the nervous system | G53 |
| 6 | Nerve root and plexus disorders | Diseases of the nervous system | G54 |
| 6 | Nerve root and plexus compressions in diseases classified elsewhere | Diseases of the nervous system | G55 |
| 6 | Mononeuropathies of upper limb | Diseases of the nervous system | G56 |
| 6 | Mononeuropathies of lower limb | Diseases of the nervous system | G57 |
| 6 | Other mononeuropathies | Diseases of the nervous system | G58 |
| 6 | Mononeuropathy in diseases classified elsewhere | Diseases of the nervous system | G59 |
| 6 | Hereditary and idiopathic neuropathy | Diseases of the nervous system | G60 |
| 6 | Inflammatory polyneuropathy | Diseases of the nervous system | G61 |
| 6 | Other polyneuropathies | Diseases of the nervous system | G62 |
| 6 | Polyneuropathy in diseases classified elsewhere | Diseases of the nervous system | G63 |
| 6 | Other disorders of peripheral nervous system | Diseases of the nervous system | G64 |
| 6 | Myasthenia gravis and other myoneural disorders | Diseases of the nervous system | G70 |
| 6 | Primary disorders of muscles | Diseases of the nervous system | G71 |
| 6 | Other myopathies | Diseases of the nervous system | G72 |
| 6 | Disorders of myoneural junction and muscle in diseases classified | Diseases of the nervous system | G73 |
| 6 | Cerebral palsy | Diseases of the nervous system | G80 |
| 6 | Hemiplegia | Diseases of the nervous system | G81 |
| 6 | Paraplegia and tetraplegia | Diseases of the nervous system | G82 |
| 6 | Other paralytic syndromes | Diseases of the nervous system | G83 |
| 6 | Disorders of autonomic nervous system | Diseases of the nervous system | G90 |
| 6 | Hydrocephalus | Diseases of the nervous system | G91 |
| 6 | Toxic encephalopathy | Diseases of the nervous system | G92 |
| 6 | Other disorders of brain | Diseases of the nervous system | G93 |
| 6 | Other diseases of spinal cord | Diseases of the nervous system | G95 |
| 6 | Other disorders of central nervous system | Diseases of the nervous system | G96 |
| 6 | Postprocedural disorders of nervous system, not elsewhere classified | Diseases of the nervous system | G97 |
| 6 | Other disorders of nervous system, not elsewhere classified | Diseases of the nervous system | G98 |
| 6 | Other disorders of nervous system in diseases classified elsewhere | Diseases of the nervous system | G99 |
| 7 | Hordeolum and chalazion | Diseases of the eye and adnexa | H00 |
| 7 | Other inflammation of eyelid | Diseases of the eye and adnexa | H01 |
| 7 | Other disorders of eyelid | Diseases of the eye and adnexa | H02 |
| 7 | Disorders of eyelid in diseases classified elsewhere | Diseases of the eye and adnexa | H03 |
| 7 | Disorders of lacrimal system | Diseases of the eye and adnexa | H04 |
| 7 | Disorders of orbit | Diseases of the eye and adnexa | H05 |
| 7 | Disorders of lacrimal system and orbit in diseases classified elsewhere | Diseases of the eye and adnexa | H06 |
| 7 | Conjunctivitis | Diseases of the eye and adnexa | H10 |
| 7 | Other disorders of conjunctiva | Diseases of the eye and adnexa | H11 |
| 7 | Disorders of conjunctiva in diseases classified elsewhere | Diseases of the eye and adnexa | H13 |
| 7 | Disorders of sclera | Diseases of the eye and adnexa | H15 |
| 7 | Keratitis | Diseases of the eye and adnexa | H16 |
| 7 | Corneal scars and opacities | Diseases of the eye and adnexa | H17 |
| 7 | Other disorders of cornea | Diseases of the eye and adnexa | H18 |
| 7 | Disorders of sclera and cornea in diseases classified elsewhere | Diseases of the eye and adnexa | H19 |
| 7 | Iridocyclitis | Diseases of the eye and adnexa | H20 |
| 7 | Other disorders of iris and ciliary body | Diseases of the eye and adnexa | H21 |
| 7 | Disorders of iris and ciliary body in diseases classified elsewhere | Diseases of the eye and adnexa | H22 |
| 7 | Senile cataract | Diseases of the eye and adnexa | H25 |
| 7 | Other cataract | Diseases of the eye and adnexa | H26 |
| 7 | Other disorders of lens | Diseases of the eye and adnexa | H27 |
| 7 | Cataract and other disorders of lens in diseases classified elsewhere | Diseases of the eye and adnexa | H28 |
| 7 | Chorioretinal inflammation | Diseases of the eye and adnexa | H30 |
| 7 | Other disorders of choroid | Diseases of the eye and adnexa | H31 |
| 7 | Chorioretinal disorders in diseases classified elsewhere | Diseases of the eye and adnexa | H32 |
| 7 | Retinal detachments and breaks | Diseases of the eye and adnexa | H33 |
| 7 | Retinal vascular occlusions | Diseases of the eye and adnexa | H34 |
| 7 | Other retinal disorders | Diseases of the eye and adnexa | H35 |
| 7 | Retinal disorders in diseases classified elsewhere | Diseases of the eye and adnexa | H36 |
| 7 | Glaucoma | Diseases of the eye and adnexa | H40 |
| 7 | Glaucoma in diseases classified elsewhere | Diseases of the eye and adnexa | H42 |
| 7 | Disorders of vitreous body | Diseases of the eye and adnexa | H43 |
| 7 | Disorders of globe | Diseases of the eye and adnexa | H44 |
| 7 | Disorders of vitreous body and globe in diseases classified elsewhere | Diseases of the eye and adnexa | H45 |
| 7 | Optic neuritis | Diseases of the eye and adnexa | H46 |
| 7 | Other disorders of optic [2nd] nerve and visual pathways | Diseases of the eye and adnexa | H47 |
| 7 | Disorders of optic [2nd] nerve and visual pathways in diseases classified | Diseases of the eye and adnexa | H48 |
| 7 | Paralytic strabismus | Diseases of the eye and adnexa | H49 |
| 7 | Other strabismus | Diseases of the eye and adnexa | H50 |
| 7 | Other disorders of binocular movement | Diseases of the eye and adnexa | H51 |
| 7 | Disorders of refraction and accommodation | Diseases of the eye and adnexa | H52 |
| 7 | Visual disturbances | Diseases of the eye and adnexa | H53 |
| 7 | Visual impairment including blindness (binocular or monocular) | Diseases of the eye and adnexa | H54 |
| 7 | Nystagmus and other irregular eye movements | Diseases of the eye and adnexa | H55 |
| 7 | Other disorders of eye and adnexa | Diseases of the eye and adnexa | H57 |
| 7 | Postprocedural disorders of eye and adnexa, not elsewhere classified | Diseases of the eye and adnexa | H59 |
| 8 | Otitis externa | Diseases of the ear and mastoid process | H60 |
| 8 | Other disorders of external ear | Diseases of the ear and mastoid process | H61 |
| 8 | Disorders of external ear in diseases classified elsewhere | Diseases of the ear and mastoid process | H62 |
| 8 | Nonsuppurative otitis media | Diseases of the ear and mastoid process | H65 |
| 8 | Suppurative and unspecified otitis media | Diseases of the ear and mastoid process | H66 |
| 8 | Eustachian salpingitis and obstruction | Diseases of the ear and mastoid process | H68 |
| 8 | Other disorders of Eustachian tube | Diseases of the ear and mastoid process | H69 |
| 8 | Mastoiditis and related conditions | Diseases of the ear and mastoid process | H70 |
| 8 | Cholesteatoma of middle ear | Diseases of the ear and mastoid process | H71 |
| 8 | Perforation of tympanic membrane | Diseases of the ear and mastoid process | H72 |
| 8 | Other disorders of tympanic membrane | Diseases of the ear and mastoid process | H73 |
| 8 | Other disorders of middle ear and mastoid | Diseases of the ear and mastoid process | H74 |
| 8 | Otosclerosis | Diseases of the ear and mastoid process | H80 |
| 8 | Disorders of vestibular function | Diseases of the ear and mastoid process | H81 |
| 8 | Vertiginous syndromes in diseases classified elsewhere | Diseases of the ear and mastoid process | H82 |
| 8 | Other diseases of inner ear | Diseases of the ear and mastoid process | H83 |
| 8 | Conductive and sensorineural hearing loss | Diseases of the ear and mastoid process | H90 |
| 8 | Other hearing loss | Diseases of the ear and mastoid process | H91 |
| 8 | Otalgia and effusion of ear | Diseases of the ear and mastoid process | H92 |
| 8 | Other disorders of ear, not elsewhere classified | Diseases of the ear and mastoid process | H93 |
| 8 | Postprocedural disorders of ear and mastoid process, not elsewhere | Diseases of the ear and mastoid process | H95 |
| 9 | Rheumatic fever with heart involvement | Diseases of the circulatory system | I01 |
| 9 | Rheumatic mitral valve diseases | Diseases of the circulatory system | I05 |
| 9 | Rheumatic aortic valve diseases | Diseases of the circulatory system | I06 |
| 9 | Rheumatic tricuspid valve diseases | Diseases of the circulatory system | I07 |
| 9 | Multiple valve diseases | Diseases of the circulatory system | I08 |
| 9 | Other rheumatic heart diseases | Diseases of the circulatory system | I09 |
| 9 | Essential (primary) hypertension | Diseases of the circulatory system | I10 |
| 9 | Hypertensive heart disease | Diseases of the circulatory system | I11 |
| 9 | Hypertensive renal disease | Diseases of the circulatory system | I12 |
| 9 | Hypertensive heart and renal disease | Diseases of the circulatory system | I13 |
| 9 | Secondary hypertension | Diseases of the circulatory system | I15 |
| 9 | Angina pectoris | Diseases of the circulatory system | I20 |
| 9 | Acute myocardial infarction | Diseases of the circulatory system | I21 |
| 9 | Subsequent myocardial infarction | Diseases of the circulatory system | I22 |
| 9 | Certain current complications following acute myocardial infarction | Diseases of the circulatory system | I23 |
| 9 | Other acute ischaemic heart diseases | Diseases of the circulatory system | I24 |
| 9 | Chronic ischaemic heart disease | Diseases of the circulatory system | I25 |
| 9 | Pulmonary embolism | Diseases of the circulatory system | I26 |
| 9 | Other pulmonary heart diseases | Diseases of the circulatory system | I27 |
| 9 | Other diseases of pulmonary vessels | Diseases of the circulatory system | I28 |
| 9 | Acute pericarditis | Diseases of the circulatory system | I30 |
| 9 | Other diseases of pericardium | Diseases of the circulatory system | I31 |
| 9 | Acute and subacute endocarditis | Diseases of the circulatory system | I33 |
| 9 | Nonrheumatic mitral valve disorders | Diseases of the circulatory system | I34 |
| 9 | Nonrheumatic aortic valve disorders | Diseases of the circulatory system | I35 |
| 9 | Nonrheumatic tricuspid valve disorders | Diseases of the circulatory system | I36 |
| 9 | Pulmonary valve disorders | Diseases of the circulatory system | I37 |
| 9 | Endocarditis, valve unspecified | Diseases of the circulatory system | I38 |
| 9 | Endocarditis and heart valve disorders in diseases classified elsewhere | Diseases of the circulatory system | I39 |
| 9 | Acute myocarditis | Diseases of the circulatory system | I40 |
| 9 | Myocarditis in diseases classified elsewhere | Diseases of the circulatory system | I41 |
| 9 | Cardiomyopathy | Diseases of the circulatory system | I42 |
| 9 | Atrioventricular and left bundle-branch block | Diseases of the circulatory system | I44 |
| 9 | Other conduction disorders | Diseases of the circulatory system | I45 |
| 9 | Cardiac arrest | Diseases of the circulatory system | I46 |
| 9 | Paroxysmal tachycardia | Diseases of the circulatory system | I47 |
| 9 | Atrial fibrillation and flutter | Diseases of the circulatory system | I48 |
| 9 | Other cardiac arrhythmias | Diseases of the circulatory system | I49 |
| 9 | Heart failure | Diseases of the circulatory system | I50 |
| 9 | Complications and ill-defined descriptions of heart disease | Diseases of the circulatory system | I51 |
| 9 | Other heart disorders in diseases classified elsewhere | Diseases of the circulatory system | I52 |
| 9 | Subarachnoid haemorrhage | Diseases of the circulatory system | I60 |
| 9 | Intracerebral haemorrhage | Diseases of the circulatory system | I61 |
| 9 | Other nontraumatic intracranial haemorrhage | Diseases of the circulatory system | I62 |
| 9 | Cerebral infarction | Diseases of the circulatory system | I63 |
| 9 | Stroke, not specified as haemorrhage or infarction | Diseases of the circulatory system | I64 |
| 9 | Occlusion and stenosis of precerebral arteries, not resulting in cerebral | Diseases of the circulatory system | I65 |
| 9 | Occlusion and stenosis of cerebral arteries, not resulting in cerebral | Diseases of the circulatory system | I66 |
| 9 | Other cerebrovascular diseases | Diseases of the circulatory system | I67 |
| 9 | Cerebrovascular disorders in diseases classified elsewhere | Diseases of the circulatory system | I68 |
| 9 | Sequelae of cerebrovascular disease | Diseases of the circulatory system | I69 |
| 9 | Atherosclerosis | Diseases of the circulatory system | I70 |
| 9 | Aortic aneurysm and dissection | Diseases of the circulatory system | I71 |
| 9 | Other aneurysm and dissection | Diseases of the circulatory system | I72 |
| 9 | Other peripheral vascular diseases | Diseases of the circulatory system | I73 |
| 9 | Arterial embolism and thrombosis | Diseases of the circulatory system | I74 |
| 9 | Other disorders of arteries and arterioles | Diseases of the circulatory system | I77 |
| 9 | Diseases of capillaries | Diseases of the circulatory system | I78 |
| 9 | Disorders of arteries, arterioles and capillaries in diseases classified | Diseases of the circulatory system | I79 |
| 9 | Phlebitis and thrombophlebitis | Diseases of the circulatory system | I80 |
| 9 | Portal vein thrombosis | Diseases of the circulatory system | I81 |
| 9 | Other venous embolism and thrombosis | Diseases of the circulatory system | I82 |
| 9 | Varicose veins of lower extremities | Diseases of the circulatory system | I83 |
| 9 | Oesophageal varices | Diseases of the circulatory system | I85 |
| 9 | Varicose veins of other sites | Diseases of the circulatory system | I86 |
| 9 | Other disorders of veins | Diseases of the circulatory system | I84 |
| 9 | Other disorders of veins | Diseases of the circulatory system | I87 |
| 9 | Nonspecific lymphadenitis | Diseases of the circulatory system | I88 |
| 9 | Other noninfective disorders of lymphatic vessels and lymph nodes | Diseases of the circulatory system | I89 |
| 9 | Hypotension | Diseases of the circulatory system | I95 |
| 9 | Postprocedural disorders of circulatory system, not elsewhere classified | Diseases of the circulatory system | I97 |
| 9 | Other disorders of circulatory system in diseases classified elsewhere | Diseases of the circulatory system | I98 |
| 9 | Other and unspecified disorders of circulatory system | Diseases of the circulatory system | I99 |
| 10 | Acute nasopharyngitis [common cold] | Diseases of the respiratory system | J00 |
| 10 | Acute sinusitis | Diseases of the respiratory system | J01 |
| 10 | Acute pharyngitis | Diseases of the respiratory system | J02 |
| 10 | Acute tonsillitis | Diseases of the respiratory system | J03 |
| 10 | Acute laryngitis and tracheitis | Diseases of the respiratory system | J04 |
| 10 | Acute obstructive laryngitis [croup] and epiglottitis | Diseases of the respiratory system | J05 |
| 10 | Acute upper respiratory infections of multiple and unspecified sites | Diseases of the respiratory system | J06 |
| 10 | Influenza due to identified zoonotic or pandemic influenza virus | Diseases of the respiratory system | J09 |
| 10 | influenza due to identified seasonal influenza virus | Diseases of the respiratory system | J10 |
| 10 | Influenza, virus not identified | Diseases of the respiratory system | J11 |
| 10 | Viral pneumonia, not elsewhere classified | Diseases of the respiratory system | J12 |
| 10 | Pneumonia due to Streptococcus pneumoniae | Diseases of the respiratory system | J13 |
| 10 | Pneumonia due to Haemophilus influenzae | Diseases of the respiratory system | J14 |
| 10 | Bacterial pneumonia, not elsewhere classified | Diseases of the respiratory system | J15 |
| 10 | Pneumonia due to other infectious organisms, not elsewhere classified | Diseases of the respiratory system | J16 |
| 10 | Pneumonia in diseases classified elsewhere | Diseases of the respiratory system | J17 |
| 10 | Pneumonia, organism unspecified | Diseases of the respiratory system | J18 |
| 10 | Acute bronchitis | Diseases of the respiratory system | J20 |
| 10 | Acute bronchiolitis | Diseases of the respiratory system | J21 |
| 10 | Unspecified acute lower respiratory infection | Diseases of the respiratory system | J22 |
| 10 | Vasomotor and allergic rhinitis | Diseases of the respiratory system | J30 |
| 10 | Chronic rhinitis, nasopharyngitis and pharyngitis | Diseases of the respiratory system | J31 |
| 10 | Chronic sinusitis | Diseases of the respiratory system | J32 |
| 10 | Nasal polyp | Diseases of the respiratory system | J33 |
| 10 | Other disorders of nose and nasal sinuses | Diseases of the respiratory system | J34 |
| 10 | Chronic diseases of tonsils and adenoids | Diseases of the respiratory system | J35 |
| 10 | Peritonsillar abscess | Diseases of the respiratory system | J36 |
| 10 | Chronic laryngitis and laryngotracheitis | Diseases of the respiratory system | J37 |
| 10 | Diseases of vocal cords and larynx, not elsewhere classified | Diseases of the respiratory system | J38 |
| 10 | Other diseases of upper respiratory tract | Diseases of the respiratory system | J39 |
| 10 | Bronchitis, not specified as acute or chronic | Diseases of the respiratory system | J40 |
| 10 | Simple and mucopurulent chronic bronchitis | Diseases of the respiratory system | J41 |
| 10 | Unspecified chronic bronchitis | Diseases of the respiratory system | J42 |
| 10 | Emphysema | Diseases of the respiratory system | J43 |
| 10 | Other chronic obstructive pulmonary disease | Diseases of the respiratory system | J44 |
| 10 | Asthma | Diseases of the respiratory system | J45 |
| 10 | Status asthmaticus | Diseases of the respiratory system | J46 |
| 10 | Bronchiectasis | Diseases of the respiratory system | J47 |
| 10 | Pneumoconiosis due to asbestos and other mineral fibres | Diseases of the respiratory system | J61 |
| 10 | Pneumoconiosis due to dust containing silica | Diseases of the respiratory system | J62 |
| 10 | Pneumoconiosis due to other inorganic dusts | Diseases of the respiratory system | J63 |
| 10 | Hypersensitivity pneumonitis due to organic dust | Diseases of the respiratory system | J67 |
| 10 | Respiratory conditions due to inhalation of chemicals, gases, fumes and | Diseases of the respiratory system | J68 |
| 10 | Pneumonitis due to solids and liquids | Diseases of the respiratory system | J69 |
| 10 | Respiratory conditions due to other external agents | Diseases of the respiratory system | J70 |
| 10 | Adult respiratory distress syndrome | Diseases of the respiratory system | J80 |
| 10 | Pulmonary oedema | Diseases of the respiratory system | J81 |
| 10 | Pulmonary eosinophilia, not elsewhere classified | Diseases of the respiratory system | J82 |
| 10 | Other interstitial pulmonary diseases | Diseases of the respiratory system | J84 |
| 10 | Abscess of lung and mediastinum | Diseases of the respiratory system | J85 |
| 10 | Pyothorax | Diseases of the respiratory system | J86 |
| 10 | Pleural effusion, not elsewhere classified | Diseases of the respiratory system | J90 |
| 10 | Pleural effusion in conditions classified elsewhere | Diseases of the respiratory system | J91 |
| 10 | Pleural plaque | Diseases of the respiratory system | J92 |
| 10 | Pneumothorax | Diseases of the respiratory system | J93 |
| 10 | Other pleural conditions | Diseases of the respiratory system | J94 |
| 10 | Postprocedural respiratory disorders, not elsewhere classified | Diseases of the respiratory system | J95 |
| 10 | Respiratory failure, not elsewhere classified | Diseases of the respiratory system | J96 |
| 10 | Other respiratory disorders | Diseases of the respiratory system | J98 |
| 10 | Respiratory disorders in diseases classified elsewhere | Diseases of the respiratory system | J99 |
| 11 | Disorders of tooth development and eruption | Diseases of the digestive system | K00 |
| 11 | Embedded and impacted teeth | Diseases of the digestive system | K01 |
| 11 | Dental caries | Diseases of the digestive system | K02 |
| 11 | Other diseases of hard tissues of teeth | Diseases of the digestive system | K03 |
| 11 | Diseases of pulp and periapical tissues | Diseases of the digestive system | K04 |
| 11 | Gingivitis and periodontal diseases | Diseases of the digestive system | K05 |
| 11 | Other disorders of gingiva and edentulous alveolar ridge | Diseases of the digestive system | K06 |
| 11 | Dentofacial anomalies [including malocclusion] | Diseases of the digestive system | K07 |
| 11 | Other disorders of teeth and supporting structures | Diseases of the digestive system | K08 |
| 11 | Cysts of oral region, not elsewhere classified | Diseases of the digestive system | K09 |
| 11 | Other diseases of jaws | Diseases of the digestive system | K10 |
| 11 | Diseases of salivary glands | Diseases of the digestive system | K11 |
| 11 | Stomatitis and related lesions | Diseases of the digestive system | K12 |
| 11 | Other diseases of lip and oral mucosa | Diseases of the digestive system | K13 |
| 11 | Diseases of tongue | Diseases of the digestive system | K14 |
| 11 | Oesophagitis | Diseases of the digestive system | K20 |
| 11 | Gastro-oesophageal reflux disease | Diseases of the digestive system | K21 |
| 11 | Other diseases of oesophagus | Diseases of the digestive system | K22 |
| 11 | Gastric ulcer | Diseases of the digestive system | K25 |
| 11 | Duodenal ulcer | Diseases of the digestive system | K26 |
| 11 | Peptic ulcer, site unspecified | Diseases of the digestive system | K27 |
| 11 | Gastrojejunal ulcer | Diseases of the digestive system | K28 |
| 11 | Gastritis and duodenitis | Diseases of the digestive system | K29 |
| 11 | Functional dyspepsia | Diseases of the digestive system | K30 |
| 11 | Other diseases of stomach and duodenum | Diseases of the digestive system | K31 |
| 11 | Acute appendicitis | Diseases of the digestive system | K35 |
| 11 | Other appendicitis | Diseases of the digestive system | K36 |
| 11 | Unspecified appendicitis | Diseases of the digestive system | K37 |
| 11 | Other diseases of appendix | Diseases of the digestive system | K38 |
| 11 | Inguinal hernia | Diseases of the digestive system | K40 |
| 11 | Femoral hernia | Diseases of the digestive system | K41 |
| 11 | Umbilical hernia | Diseases of the digestive system | K42 |
| 11 | Ventral hernia | Diseases of the digestive system | K43 |
| 11 | Diaphragmatic hernia | Diseases of the digestive system | K44 |
| 11 | Other abdominal hernia | Diseases of the digestive system | K45 |
| 11 | Unspecified abdominal hernia | Diseases of the digestive system | K46 |
| 11 | Crohn’s disease [regional enteritis] | Diseases of the digestive system | K50 |
| 11 | Ulcerative colitis | Diseases of the digestive system | K51 |
| 11 | Other noninfective gastroenteritis and colitis | Diseases of the digestive system | K52 |
| 11 | Vascular disorders of intestine | Diseases of the digestive system | K55 |
| 11 | Paralytic ileus and intestinal obstruction without hernia | Diseases of the digestive system | K56 |
| 11 | Diverticular disease of intestine | Diseases of the digestive system | K57 |
| 11 | Irritable bowel syndrome | Diseases of the digestive system | K58 |
| 11 | Other functional intestinal disorders | Diseases of the digestive system | K59 |
| 11 | Fissure and fistula of anal and rectal regions | Diseases of the digestive system | K60 |
| 11 | Abscess of anal and rectal regions | Diseases of the digestive system | K61 |
| 11 | Other diseases of anus and rectum | Diseases of the digestive system | K62 |
| 11 | Other diseases of intestine | Diseases of the digestive system | K63 |
| 11 | Haemorrhoids and perianal venous thrombosis | Diseases of the digestive system | K64 |
| 11 | Peritonitis | Diseases of the digestive system | K65 |
| 11 | Other disorders of peritoneum | Diseases of the digestive system | K66 |
| 11 | Alcoholic liver disease | Diseases of the digestive system | K70 |
| 11 | Toxic liver disease | Diseases of the digestive system | K71 |
| 11 | Hepatic failure, not elsewhere classified | Diseases of the digestive system | K72 |
| 11 | Chronic hepatitis, not elsewhere classified | Diseases of the digestive system | K73 |
| 11 | Fibrosis and cirrhosis of liver | Diseases of the digestive system | K74 |
| 11 | Other inflammatory liver diseases | Diseases of the digestive system | K75 |
| 11 | Other diseases of liver | Diseases of the digestive system | K76 |
| 11 | Liver disorders in diseases classified elsewhere | Diseases of the digestive system | K77 |
| 11 | Cholelithiasis | Diseases of the digestive system | K80 |
| 11 | Cholecystitis | Diseases of the digestive system | K81 |
| 11 | Other diseases of gallbladder | Diseases of the digestive system | K82 |
| 11 | Other diseases of biliary tract | Diseases of the digestive system | K83 |
| 11 | Acute pancreatitis | Diseases of the digestive system | K85 |
| 11 | Other diseases of pancreas | Diseases of the digestive system | K86 |
| 11 | Disorders of gallbladder, biliary tract and pancreas in diseases classified | Diseases of the digestive system | K87 |
| 11 | Intestinal malabsorption | Diseases of the digestive system | K90 |
| 11 | Postprocedural disorders of digestive system, not elsewhere classified | Diseases of the digestive system | K91 |
| 11 | Other diseases of digestive system | Diseases of the digestive system | K92 |
| 13 | Pyogenic arthritis | Diseases of the musculoskeletal system an | M00 |
| 13 | Direct infections of joint in infectious and parasitic diseases classified | Diseases of the musculoskeletal system an | M01 |
| 13 | Reactive arthropathies | Diseases of the musculoskeletal system an | M02 |
| 13 | Postinfective and reactive arthropathies in diseases classified elsewhere | Diseases of the musculoskeletal system an | M03 |
| 13 | Seropositive rheumatoid arthritis | Diseases of the musculoskeletal system an | M05 |
| 13 | Other rheumatoid arthritis | Diseases of the musculoskeletal system an | M06 |
| 13 | Psoriatic and enteropathic arthropathies | Diseases of the musculoskeletal system an | M07 |
| 13 | Juvenile arthritis | Diseases of the musculoskeletal system an | M08 |
| 13 | Juvenile arthritis in diseases classified elsewhere | Diseases of the musculoskeletal system an | M09 |
| 13 | Gout | Diseases of the musculoskeletal system an | M10 |
| 13 | Other crystal arthropathies | Diseases of the musculoskeletal system an | M11 |
| 13 | Other specific arthropathies | Diseases of the musculoskeletal system an | M12 |
| 13 | Other arthritis | Diseases of the musculoskeletal system an | M13 |
| 13 | Arthropathies in other diseases classified elsewhere | Diseases of the musculoskeletal system an | M14 |
| 13 | Polyarthrosis | Diseases of the musculoskeletal system an | M15 |
| 13 | Coxarthrosis [arthrosis of hip] | Diseases of the musculoskeletal system an | M16 |
| 13 | Gonarthrosis [arthrosis of knee] | Diseases of the musculoskeletal system an | M17 |
| 13 | Arthrosis of first carpometacarpal joint | Diseases of the musculoskeletal system an | M18 |
| 13 | Other arthrosis | Diseases of the musculoskeletal system an | M19 |
| 13 | Acquired deformities of fingers and toes | Diseases of the musculoskeletal system an | M20 |
| 13 | Other acquired deformities of limbs | Diseases of the musculoskeletal system an | M21 |
| 13 | Disorders of patella | Diseases of the musculoskeletal system an | M22 |
| 13 | Internal derangement of knee | Diseases of the musculoskeletal system an | M23 |
| 13 | Other specific joint derangements | Diseases of the musculoskeletal system an | M24 |
| 13 | Other joint disorders, not elsewhere classified | Diseases of the musculoskeletal system an | M25 |
| 13 | Polyarteritis nodosa and related conditions | Diseases of the musculoskeletal system an | M30 |
| 13 | Other necrotizing vasculopathies | Diseases of the musculoskeletal system an | M31 |
| 13 | Systemic lupus erythematosus | Diseases of the musculoskeletal system an | M32 |
| 13 | Dermatopolymyositis | Diseases of the musculoskeletal system an | M33 |
| 13 | Systemic sclerosis | Diseases of the musculoskeletal system an | M34 |
| 13 | Other systemic involvement of connective tissue | Diseases of the musculoskeletal system an | M35 |
| 13 | Systemic disorders of connective tissue in diseases classified elsewhere | Diseases of the musculoskeletal system an | M36 |
| 13 | Kyphosis and lordosis | Diseases of the musculoskeletal system an | M40 |
| 13 | Scoliosis | Diseases of the musculoskeletal system an | M41 |
| 13 | Spinal osteochondrosis | Diseases of the musculoskeletal system an | M42 |
| 13 | Other deforming dorsopathies | Diseases of the musculoskeletal system an | M43 |
| 13 | Ankylosing spondylitis | Diseases of the musculoskeletal system an | M45 |
| 13 | Other inflammatory spondylopathies | Diseases of the musculoskeletal system an | M46 |
| 13 | Spondylosis | Diseases of the musculoskeletal system an | M47 |
| 13 | Other spondylopathies | Diseases of the musculoskeletal system an | M48 |
| 13 | Spondylopathies in diseases classified elsewhere | Diseases of the musculoskeletal system an | M49 |
| 13 | Cervical disc disorders | Diseases of the musculoskeletal system an | M50 |
| 13 | Other intervertebral disc disorders | Diseases of the musculoskeletal system an | M51 |
| 13 | Other dorsopathies, not elsewhere classified | Diseases of the musculoskeletal system an | M53 |
| 13 | Dorsalgia | Diseases of the musculoskeletal system an | M54 |
| 13 | Myositis | Diseases of the musculoskeletal system an | M60 |
| 13 | Calcification and ossification of muscle | Diseases of the musculoskeletal system an | M61 |
| 13 | Other disorders of muscle | Diseases of the musculoskeletal system an | M62 |
| 13 | Synovitis and tenosynovitis | Diseases of the musculoskeletal system an | M65 |
| 13 | Spontaneous rupture of synovium and tendon | Diseases of the musculoskeletal system an | M66 |
| 13 | Other disorders of synovium and tendon | Diseases of the musculoskeletal system an | M67 |
| 13 | Disorders of synovium and tendon in diseases classified elsewhere | Diseases of the musculoskeletal system an | M68 |
| 13 | Soft tissue disorders related to use, overuse and pressure | Diseases of the musculoskeletal system an | M70 |
| 13 | Other bursopathies | Diseases of the musculoskeletal system an | M71 |
| 13 | Fibroblastic disorders | Diseases of the musculoskeletal system an | M72 |
| 13 | Soft tissue disorders in diseases classified elsewhere | Diseases of the musculoskeletal system an | M73 |
| 13 | Shoulder lesions | Diseases of the musculoskeletal system an | M75 |
| 13 | Enthesopathies of lower limb, excluding foot | Diseases of the musculoskeletal system an | M76 |
| 13 | Other enthesopathies | Diseases of the musculoskeletal system an | M77 |
| 13 | Other soft tissue disorders, not elsewhere classified | Diseases of the musculoskeletal system an | M79 |
| 13 | Osteoporosis with pathological fracture | Diseases of the musculoskeletal system an | M80 |
| 13 | Osteoporosis without pathological fracture | Diseases of the musculoskeletal system an | M81 |
| 13 | Osteoporosis in diseases classified elsewhere | Diseases of the musculoskeletal system an | M82 |
| 13 | Adult osteomalacia | Diseases of the musculoskeletal system an | M83 |
| 13 | Disorders of continuity of bone | Diseases of the musculoskeletal system an | M84 |
| 13 | Other disorders of bone density and structure | Diseases of the musculoskeletal system an | M85 |
| 13 | Osteomyelitis | Diseases of the musculoskeletal system an | M86 |
| 13 | Osteonecrosis | Diseases of the musculoskeletal system an | M87 |
| 13 | Paget disease of bone [osteitis deformans] | Diseases of the musculoskeletal system an | M88 |
| 13 | Other disorders of bone | Diseases of the musculoskeletal system an | M89 |
| 13 | Osteopathies in diseases classified elsewhere | Diseases of the musculoskeletal system an | M90 |
| 13 | Juvenile osteochondrosis of hip and pelvis | Diseases of the musculoskeletal system an | M91 |
| 13 | Other juvenile osteochondrosis | Diseases of the musculoskeletal system an | M92 |
| 13 | Other osteochondropathies | Diseases of the musculoskeletal system an | M93 |
| 13 | Other disorders of cartilage | Diseases of the musculoskeletal system an | M94 |
| 13 | Other acquired deformities of musculoskeletal system and connective | Diseases of the musculoskeletal system an | M95 |
| 13 | Postprocedural musculoskeletal disorders, not elsewhere classified | Diseases of the musculoskeletal system an | M96 |
| 13 | Biomechanical lesions, not elsewhere classified | Diseases of the musculoskeletal system an | M99 |
| 14 | Acute nephritic syndrome | Diseases of the genitourinary system | N00 |
| 14 | Rapidly progressive nephritic syndrome | Diseases of the genitourinary system | N01 |
| 14 | Recurrent and persistent haematuria | Diseases of the genitourinary system | N02 |
| 14 | Chronic nephritic syndrome | Diseases of the genitourinary system | N03 |
| 14 | Nephrotic syndrome | Diseases of the genitourinary system | N04 |
| 14 | Unspecified nephritic syndrome | Diseases of the genitourinary system | N05 |
| 14 | Isolated proteinuria with specified morphological lesion | Diseases of the genitourinary system | N06 |
| 14 | Hereditary nephropathy, not elsewhere classified | Diseases of the genitourinary system | N07 |
| 14 | Glomerular disorders in diseases classified elsewhere | Diseases of the genitourinary system | N08 |
| 14 | Acute tubulo-interstitial nephritis | Diseases of the genitourinary system | N10 |
| 14 | Chronic tubulo-interstitial nephritis | Diseases of the genitourinary system | N11 |
| 14 | Tubulo-interstitial nephritis, not specified as acute or chronic | Diseases of the genitourinary system | N12 |
| 14 | Obstructive and reflux uropathy | Diseases of the genitourinary system | N13 |
| 14 | Drug- and heavy-metal-induced tubulo-interstitial and tubular conditions | Diseases of the genitourinary system | N14 |
| 14 | Other renal tubulo-interstitial diseases | Diseases of the genitourinary system | N15 |
| 14 | Renal tubulo-interstitial disorders in diseases classified elsewhere | Diseases of the genitourinary system | N16 |
| 14 | Acute renal failure | Diseases of the genitourinary system | N17 |
| 14 | Chronic kidney disease | Diseases of the genitourinary system | N18 |
| 14 | Unspecified kidney failure | Diseases of the genitourinary system | N19 |
| 14 | Calculus of kidney and ureter | Diseases of the genitourinary system | N20 |
| 14 | Calculus of lower urinary tract | Diseases of the genitourinary system | N21 |
| 14 | Unspecified renal colic | Diseases of the genitourinary system | N23 |
| 14 | Disorders resulting from impaired renal tubular function | Diseases of the genitourinary system | N25 |
| 14 | Unspecified contracted kidney | Diseases of the genitourinary system | N26 |
| 14 | Small kidney of unknown cause | Diseases of the genitourinary system | N27 |
| 14 | Other disorders of kidney and ureter, not elsewhere classified | Diseases of the genitourinary system | N28 |
| 14 | Other disorders of kidney and ureter in diseases classified elsewhere | Diseases of the genitourinary system | N29 |
| 14 | Cystitis | Diseases of the genitourinary system | N30 |
| 14 | Neuromuscular dysfunction of bladder, not elsewhere classified | Diseases of the genitourinary system | N31 |
| 14 | Other disorders of bladder | Diseases of the genitourinary system | N32 |
| 14 | Bladder disorders in diseases classified elsewhere | Diseases of the genitourinary system | N33 |
| 14 | Urethritis and urethral syndrome | Diseases of the genitourinary system | N34 |
| 14 | Urethral stricture | Diseases of the genitourinary system | N35 |
| 14 | Other disorders of urethra | Diseases of the genitourinary system | N36 |
| 14 | Other disorders of urinary system | Diseases of the genitourinary system | N39 |
| 14 | Hyperplasia of prostate | Diseases of the genitourinary system | N40 |
| 14 | Inflammatory diseases of prostate | Diseases of the genitourinary system | N41 |
| 14 | Other disorders of prostate | Diseases of the genitourinary system | N42 |
| 14 | Hydrocele and spermatocele | Diseases of the genitourinary system | N43 |
| 14 | Torsion of testis | Diseases of the genitourinary system | N44 |
| 14 | Orchitis and epididymitis | Diseases of the genitourinary system | N45 |
| 14 | Male infertility | Diseases of the genitourinary system | N46 |
| 14 | Redundant prepuce, phimosis and paraphimosis | Diseases of the genitourinary system | N47 |
| 14 | Other disorders of penis | Diseases of the genitourinary system | N48 |
| 14 | Inflammatory disorders of male genital organs, not elsewhere classified | Diseases of the genitourinary system | N49 |
| 14 | Other disorders of male genital organs | Diseases of the genitourinary system | N50 |
| 14 | Disorders of male genital organs in diseases classified elsewhere | Diseases of the genitourinary system | N51 |
| 14 | Benign mammary dysplasia | Diseases of the genitourinary system | N60 |
| 14 | Inflammatory disorders of breast | Diseases of the genitourinary system | N61 |
| 14 | Hypertrophy of breast | Diseases of the genitourinary system | N62 |
| 14 | Unspecified lump in breast | Diseases of the genitourinary system | N63 |
| 14 | Other disorders of breast | Diseases of the genitourinary system | N64 |
| 14 | Salpingitis and oophoritis | Diseases of the genitourinary system | N70 |
| 14 | Inflammatory disease of uterus, except cervix | Diseases of the genitourinary system | N71 |
| 14 | Inflammatory disease of cervix uteri | Diseases of the genitourinary system | N72 |
| 14 | Other female pelvic inflammatory diseases | Diseases of the genitourinary system | N73 |
| 14 | Female pelvic inflammatory disorders in diseases classified elsewhere | Diseases of the genitourinary system | N74 |
| 14 | Diseases of Bartholin gland | Diseases of the genitourinary system | N75 |
| 14 | Other inflammation of vagina and vulva | Diseases of the genitourinary system | N76 |
| 14 | Vulvovaginal ulceration and inflammation in diseases classified elsewhere | Diseases of the genitourinary system | N77 |
| 14 | Endometriosis | Diseases of the genitourinary system | N80 |
| 14 | Female genital prolapse | Diseases of the genitourinary system | N81 |
| 14 | Fistulae involving female genital tract | Diseases of the genitourinary system | N82 |
| 14 | Noninflammatory disorders of ovary, fallopian tube and broad ligament | Diseases of the genitourinary system | N83 |
| 14 | Polyp of female genital tract | Diseases of the genitourinary system | N84 |
| 14 | Other noninflammatory disorders of uterus, except cervix | Diseases of the genitourinary system | N85 |
| 14 | Erosion and ectropion of cervix uteri | Diseases of the genitourinary system | N86 |
| 14 | Dysplasia of cervix uteri | Diseases of the genitourinary system | N87 |
| 14 | Other noninflammatory disorders of cervix uteri | Diseases of the genitourinary system | N88 |
| 14 | Other noninflammatory disorders of vagina | Diseases of the genitourinary system | N89 |
| 14 | Other noninflammatory disorders of vulva and perineum | Diseases of the genitourinary system | N90 |
| 14 | Absent, scanty and rare menstruation | Diseases of the genitourinary system | N91 |
| 14 | Excessive, frequent and irregular menstruation | Diseases of the genitourinary system | N92 |
| 14 | Other abnormal uterine and vaginal bleeding | Diseases of the genitourinary system | N93 |
| 14 | Pain and other conditions associated with female genital organs and | Diseases of the genitourinary system | N94 |
| 14 | Menopausal and other perimenopausal disorders | Diseases of the genitourinary system | N95 |
| 14 | Habitual aborter | Diseases of the genitourinary system | N96 |
| 14 | Female infertility | Diseases of the genitourinary system | N97 |
| 14 | Complications associated with artificial fertilization | Diseases of the genitourinary system | N98 |
| 14 | Postprocedural disorders of genitourinary system, not elsewhere classified | Diseases of the genitourinary system | N99 |

**Supplement 2 – Table 2. Results from main analysis presented in tabular format.**

| **Category** | **Incidence rate ratio (95% CI)** | **Pvalue** | **Events** | **Person-years** | **FDR** |
| --- | --- | --- | --- | --- | --- |
| Other Salmonella infections | 4.3(0.9-19.4) | 0.061489627 | 3 | 8770 | 0.136755617 |
| Other bacterial intestinal infections | 3.7(2.2-6.1) | 9.02994E-07 | 23 | 8690 | 1.09306E-05 |
| Other protozoal intestinal diseases | 3(0.3-31) | 0.364852642 | 1 | 8790 | 0.541854045 |
| Viral and other specified intestinal infections | 3(1.7-5.3) | 0.000238603 | 17 | 8730 | 0.001494025 |
| Other gastroenteritis and colitis of infectious and unspecified origin | 3.6(2.5-5) | 1.92751E-13 | 53 | 8430 | 5.22997E-12 |
| Listeriosis | 7.7(0.5-126.7) | 0.1522613 | 1 | 8790 | 0.285577646 |
| Streptococcal sepsis | 4.6(2.4-9) | 8.18164E-06 | 15 | 8760 | 7.74402E-05 |
| Other sepsis | 3.5(2.7-4.7) | 2.70311E-18 | 76 | 8560 | 1.57167E-16 |
| Erysipelas | 2.4(1.7-3.3) | 5.95456E-07 | 47 | 8540 | 8.07836E-06 |
| Bacterial infection of unspecified site | 2.8(1.8-4.3) | 7.7604E-06 | 28 | 8720 | 7.5202E-05 |
| Anogenital herpesviral [herpes simplex] infection | 2.9(0.9-9.7) | 0.081419195 | 4 | 8750 | 0.171697474 |
| Other predominantly sexually transmitted diseases, not elsewhere classified | 0.6(0.1-2.7) | 0.546230521 | 2 | 8770 | 0.690421808 |
| Other spirochaetal infections | 1.1(0.4-3.4) | 0.800651374 | 4 | 8720 | 0.873633 |
| Other viral haemorrhagic fevers, not elsewhere classified | 1.9(0.2-18.3) | 0.581219989 | 1 | 8790 | 0.706138912 |
| Herpesviral [herpes simplex] infections | 2.8(1.4-5.8) | 0.004977457 | 11 | 8710 | 0.018250676 |
| Varicella [chickenpox] | 5.5(0.8-39.3) | 0.086159503 | 2 | 8790 | 0.179830348 |
| Zoster [herpes zoster] | 2.8(1.7-4.7) | 0.000107557 | 21 | 8670 | 0.000729596 |
| Viral warts | 2.4(1.2-4.9) | 0.013884954 | 11 | 8700 | 0.041860565 |
| Other acute viral hepatitis | 5.8(0.4-92.1) | 0.212713418 | 1 | 8780 | 0.36223582 |
| Chronic viral hepatitis | 1(0.3-3.4) | 0.997231263 | 3 | 8730 | 0.997231263 |
| Human immunodeficiency virus [HIV] disease resulting in infectious | 5.7(0.4-88.3) | 0.211183982 | 1 | 8790 | 0.361198273 |
| Cytomegaloviral disease | 8.1(2.6-25.4) | 0.000372133 | 7 | 8760 | 0.002163688 |
| Infectious mononucleosis | 2(0.2-19.2) | 0.56260053 | 1 | 8780 | 0.698104926 |
| Viral conjunctivitis | 0.9(0.1-7.8) | 0.957250903 | 1 | 8780 | 0.966752153 |
| Viral infection of unspecified site | 5.3(3.6-7.8) | 0 | 50 | 8490 | 0 |
| Dermatophytosis | 1.8(0.8-4.1) | 0.137378998 | 8 | 8740 | 0.266253581 |
| Other superficial mycoses | 3.8(0.6-22.8) | 0.146279237 | 2 | 8780 | 0.278203969 |
| Candidiasis | 2.4(1.7-3.5) | 1.26034E-06 | 42 | 8590 | 1.42774E-05 |
| Scabies | 1.5(0.2-14.5) | 0.701190216 | 1 | 8780 | 0.803899769 |
| Sequelae of poliomyelitis | 3.4(0.8-14.1) | 0.092537433 | 3 | 8770 | 0.191181397 |
| Streptococcus and Staphylococcus as the cause of diseases classified | 3.2(2.2-4.7) | 1.24919E-09 | 41 | 8640 | 2.11842E-08 |
| Other specified bacterial agents as the cause of diseases classified | 2.1(1.5-2.9) | 1.34545E-05 | 47 | 8620 | 0.00011651 |
| Viral agents as the cause of diseases classified to other chapters | 9.6(2.3-39.5) | 0.001730263 | 5 | 8780 | 0.008094448 |
| Other specified infectious agents as the cause of diseases classified | 1.6(0.3-8) | 0.539513868 | 2 | 8780 | 0.68834528 |
| Other and unspecified infectious diseases | 3.7(2.6-5.2) | 7.83895E-14 | 53 | 8530 | 2.2789E-12 |
| Other hypothyroidism | 1.1(0.7-1.7) | 0.610246067 | 27 | 8530 | 0.726228506 |
| Other nontoxic goitre | 2.6(1.4-4.8) | 0.003414182 | 14 | 8650 | 0.013895719 |
| Thyrotoxicosis [hyperthyroidism] | 3.8(2.2-6.6) | 9.13124E-07 | 22 | 8640 | 1.09306E-05 |
| Thyroiditis | 1.4(0.4-5.2) | 0.569756241 | 3 | 8760 | 0.704835228 |
| Other disorders of thyroid | 3.8(0.6-22.8) | 0.148475968 | 2 | 8760 | 0.28106846 |
| Type 1 diabetes mellitus | 1.9(1.1-3.1) | 0.014792441 | 20 | 8510 | 0.043313118 |
| Type 2 diabetes mellitus | 1.4(1.1-1.8) | 0.009077983 | 75 | 8030 | 0.030284745 |
| Other specified diabetes mellitus | 7.6(1.7-34.2) | 0.008481587 | 4 | 8760 | 0.028766716 |
| Unspecified diabetes mellitus | 1.3(0.8-2.3) | 0.321742343 | 15 | 8530 | 0.492195164 |
| Other disorders of pancreatic internal secretion | 1.9(0.8-4.5) | 0.132453761 | 7 | 8760 | 0.257936272 |
| Hyperparathyroidism and other disorders of parathyroid gland | 0.3(0-2) | 0.204892379 | 1 | 8760 | 0.356372643 |
| Hyperfunction of pituitary gland | 5.7(1.4-22.7) | 0.013008367 | 4 | 8760 | 0.040726196 |
| Hypofunction and other disorders of pituitary gland | 3.8(1.1-13.4) | 0.040991935 | 4 | 8770 | 0.101113439 |
| Other disorders of adrenal gland | 7.2(1.9-26.8) | 0.003384707 | 5 | 8770 | 0.013895719 |
| Unspecified severe protein-energy malnutrition | 5.9(0.8-41.7) | 0.077523218 | 2 | 8790 | 0.166062894 |
| Unspecified protein-energy malnutrition | 2(0.6-6.2) | 0.242996935 | 4 | 8780 | 0.39559901 |
| Deficiency of other B group vitamins | 0.8(0.2-3.7) | 0.826726907 | 2 | 8790 | 0.887804357 |
| Vitamin D deficiency | 3.3(1-11.3) | 0.055332214 | 4 | 8780 | 0.12868692 |
| Deficiency of other nutrient elements | 1.2(0.1-10.5) | 0.84725128 | 1 | 8790 | 0.891036875 |
| Obesity | 0.7(0.4-1.2) | 0.183490748 | 13 | 8560 | 0.327768199 |
| Disorders of lipoprotein metabolism and other lipidaemias | 1.2(0.9-1.6) | 0.246701019 | 57 | 8120 | 0.396866857 |
| Disorders of mineral metabolism | 1(0.3-3.3) | 0.947782579 | 3 | 8750 | 0.966346489 |
| Volume depletion | 2.9(2-4.3) | 1.09863E-08 | 41 | 8680 | 1.65609E-07 |
| Other disorders of fluid, electrolyte and acid–base balance | 2.6(1.9-3.5) | 8.86648E-10 | 59 | 8560 | 1.56898E-08 |
| Other metabolic disorders | 2.8(0.5-15.5) | 0.236021623 | 2 | 8790 | 0.387341937 |
| Postprocedural endocrine and metabolic disorders, not elsewhere classified | 3.8(1.6-8.9) | 0.002214965 | 9 | 8730 | 0.00990649 |
| Dementia in Alzheimer disease (G30.-†) | 1.6(1.2-1.9) | 7.3514E-05 | 102 | 8160 | 0.000515865 |
| Vascular dementia | 0.6(0.3-1.6) | 0.340266012 | 5 | 8780 | 0.511026816 |
| Dementia in other diseases classified elsewhere | 0.5(0.1-3.7) | 0.485107946 | 1 | 8790 | 0.647340767 |
| Unspecified dementia | 1.2(0.8-1.9) | 0.415118243 | 22 | 8750 | 0.578606592 |
| Delirium, not induced by alcohol and other psychoactive substances | 0.5(0.1-3.8) | 0.510016218 | 1 | 8790 | 0.671149836 |
| Bacterial meningitis, not elsewhere classified | 5.6(1.1-28.1) | 0.036148955 | 3 | 8770 | 0.091382762 |
| Meningitis due to other and unspecified causes | 17.3(1.8-165.9) | 0.013504751 | 3 | 8770 | 0.041183879 |
| Encephalitis, myelitis and encephalomyelitis | 9(1.6-52.1) | 0.014310581 | 3 | 8780 | 0.042403179 |
| Intracranial and intraspinal abscess and granuloma | 5.8(0.8-40.7) | 0.076840628 | 2 | 8770 | 0.165471617 |
| Parkinson disease | 0.7(0.3-1.7) | 0.406535892 | 5 | 8720 | 0.570552097 |
| Secondary parkinsonism | 0.3(0-2.4) | 0.267655125 | 1 | 8790 | 0.423874069 |
| Dystonia | 1.5(0.5-4.5) | 0.466864782 | 4 | 8760 | 0.631275635 |
| Other extrapyramidal and movement disorders | 2.5(1.3-4.9) | 0.008703365 | 12 | 8660 | 0.029274956 |
| Alzheimer disease | 0.6(0.3-1.2) | 0.159581732 | 8 | 8750 | 0.294383498 |
| Other degenerative diseases of nervous system, not elsewhere classified | 2.9(0.7-11.4) | 0.124038631 | 3 | 8780 | 0.24746923 |
| Multiple sclerosis | 12(1.1-135.7) | 0.045364995 | 2 | 8770 | 0.110560199 |
| Other demyelinating diseases of central nervous system | 2.9(0.3-30.7) | 0.376569256 | 1 | 8770 | 0.550555653 |
| Epilepsy | 1(0.6-1.9) | 0.884895058 | 12 | 8670 | 0.923467407 |
| Migraine | 2.1(1.2-3.7) | 0.008329644 | 17 | 8630 | 0.028730212 |
| Other headache syndromes | 1.8(1-3.4) | 0.059211094 | 13 | 8690 | 0.133882862 |
| Transient cerebral ischaemic attacks and related syndromes | 1.4(1-2.1) | 0.060917122 | 35 | 8590 | 0.136226751 |
| Vascular syndromes of brain in cerebrovascular diseases (I60–I67†) | 11.7(1-139.2) | 0.051235079 | 2 | 8780 | 0.122662807 |
| Sleep disorders | 1.1(0.8-1.7) | 0.491161251 | 33 | 8410 | 0.651148629 |
| Disorders of trigeminal nerve | 2.4(0.9-6.9) | 0.097448493 | 5 | 8770 | 0.199601756 |
| Facial nerve disorders | 1.3(0.6-2.6) | 0.518392147 | 9 | 8720 | 0.674048589 |
| Nerve root and plexus disorders | 1(0.2-4.7) | 0.963285103 | 2 | 8780 | 0.97043821 |
| Mononeuropathies of upper limb | 0.8(0.5-1.3) | 0.463443412 | 21 | 8420 | 0.630841032 |
| Mononeuropathies of lower limb | 1.3(0.5-3.1) | 0.599364677 | 6 | 8740 | 0.71959122 |
| Hereditary and idiopathic neuropathy | 5.8(1.2-28.8) | 0.031087567 | 3 | 8770 | 0.082061821 |
| Other polyneuropathies | 3.7(2.1-6.7) | 1.19938E-05 | 18 | 8680 | 0.000108057 |
| Other disorders of peripheral nervous system | 1.9(0.2-18) | 0.588254921 | 1 | 8780 | 0.710444371 |
| Other myopathies | 1.7(0.3-8) | 0.525334867 | 2 | 8780 | 0.67734189 |
| Cerebral palsy | 1.8(0.2-17.5) | 0.593594959 | 1 | 8790 | 0.714772629 |
| Hemiplegia | 1.2(0.6-2.3) | 0.608415652 | 11 | 8750 | 0.726173521 |
| Paraplegia and tetraplegia | 1.6(0.3-7.9) | 0.549376766 | 2 | 8770 | 0.692248742 |
| Other paralytic syndromes | 1.2(0.3-5.5) | 0.833054168 | 2 | 8770 | 0.891036875 |
| Hydrocephalus | 0.8(0.2-3.7) | 0.797937661 | 2 | 8780 | 0.873633 |
| Other disorders of brain | 0.4(0.1-3.1) | 0.386690093 | 1 | 8760 | 0.554165028 |
| Hordeolum and chalazion | 0.6(0.2-2.1) | 0.45090522 | 3 | 8750 | 0.61583364 |
| Other inflammation of eyelid | 1.8(1.2-2.7) | 0.00457038 | 31 | 8560 | 0.017384529 |
| Other disorders of eyelid | 1.9(1.3-2.7) | 0.000431952 | 39 | 8500 | 0.002375735 |
| Disorders of lacrimal system | 2.6(2-3.4) | 1.46013E-12 | 80 | 8150 | 3.49573E-11 |
| Disorders of orbit | 11.5(2.8-47.2) | 0.000667357 | 6 | 8740 | 0.003527458 |
| Conjunctivitis | 3.6(2.6-4.9) | 6.83405E-15 | 60 | 8280 | 2.72029E-13 |
| Other disorders of conjunctiva | 8(5.3-12) | 0 | 54 | 8450 | 0 |
| Disorders of sclera | 0.8(0.2-3.6) | 0.786193598 | 2 | 8760 | 0.871882274 |
| Keratitis | 2.1(1.3-3.2) | 0.001624565 | 26 | 8570 | 0.007688346 |
| Corneal scars and opacities | 1.8(0.7-5) | 0.244383548 | 5 | 8740 | 0.396271331 |
| Other disorders of cornea | 2(1.2-3.4) | 0.009502457 | 19 | 8700 | 0.030939999 |
| Disorders of sclera and cornea in diseases classified elsewhere | 1.9(0.8-4.9) | 0.162737178 | 6 | 8750 | 0.298351493 |
| Iridocyclitis | 2.2(1.4-3.7) | 0.001291409 | 22 | 8630 | 0.006332573 |
| Other disorders of iris and ciliary body | 15.5(4.2-57.6) | 4.29255E-05 | 8 | 8760 | 0.000317037 |
| Senile cataract | 1.3(1.1-1.5) | 0.000993285 | 177 | 7530 | 0.004990953 |
| Other cataract | 1.3(0.9-1.7) | 0.120129147 | 57 | 8450 | 0.242042391 |
| Other disorders of lens | 1.1(0.4-3.2) | 0.852525001 | 4 | 8760 | 0.89427236 |
| Chorioretinal inflammation | 1.9(0.2-17.9) | 0.574732375 | 1 | 8780 | 0.706138912 |
| Retinal detachments and breaks | 1.2(0.6-2.2) | 0.664664372 | 11 | 8680 | 0.772909712 |
| Retinal vascular occlusions | 2.2(1.3-3.8) | 0.003878714 | 18 | 8670 | 0.015216587 |
| Other retinal disorders | 2.3(1.8-2.8) | 9.28959E-15 | 122 | 7840 | 3.15072E-13 |
| Retinal disorders in diseases classified elsewhere | 2.4(1.5-3.9) | 0.000394012 | 23 | 8510 | 0.002258635 |
| Glaucoma | 1.3(1-1.7) | 0.078621142 | 57 | 8270 | 0.167533009 |
| Disorders of vitreous body | 1.8(1.4-2.3) | 7.1253E-06 | 78 | 8120 | 7.07317E-05 |
| Disorders of globe | 1.2(0.3-4.2) | 0.77843705 | 3 | 8760 | 0.868010628 |
| Other disorders of optic [2nd] nerve and visual pathways | 2.1(0.8-5.9) | 0.149256206 | 5 | 8770 | 0.281237387 |
| Paralytic strabismus | 2.1(0.7-6.7) | 0.191101937 | 4 | 8730 | 0.336703412 |
| Other strabismus | 0.7(0.2-2.2) | 0.512844224 | 3 | 8710 | 0.671149836 |
| Disorders of refraction and accommodation | 1.1(0.6-1.8) | 0.809695276 | 18 | 8660 | 0.881138976 |
| Visual disturbances | 1.9(1.3-2.7) | 0.00029878 | 42 | 8540 | 0.001795883 |
| Visual impairment including blindness (binocular or monocular) | 2.4(1.3-4.5) | 0.005837105 | 14 | 8730 | 0.020658276 |
| Other disorders of eye and adnexa | 1.7(0.6-5.4) | 0.32837807 | 4 | 8720 | 0.496839682 |
| Otitis externa | 2.8(1.9-4.3) | 1.26287E-06 | 32 | 8550 | 1.42774E-05 |
| Other disorders of external ear | 2.1(1.5-3) | 2.44365E-05 | 43 | 8480 | 0.000198913 |
| Nonsuppurative otitis media | 2.3(1.1-4.8) | 0.02815709 | 10 | 8670 | 0.075893613 |
| Suppurative and unspecified otitis media | 3.7(1.9-7.1) | 0.00014028 | 14 | 8670 | 0.000935967 |
| Eustachian salpingitis and obstruction | 5.7(1.1-28.4) | 0.035564126 | 3 | 8780 | 0.091035216 |
| Other disorders of Eustachian tube | 3.3(1-11.2) | 0.055163075 | 4 | 8760 | 0.12868692 |
| Mastoiditis and related conditions | 1.9(0.2-18.3) | 0.577838623 | 1 | 8790 | 0.706138912 |
| Perforation of tympanic membrane | 2(0.7-5.7) | 0.169925782 | 5 | 8740 | 0.308749077 |
| Other disorders of tympanic membrane | 1.4(0.3-6.7) | 0.65716395 | 2 | 8770 | 0.767865576 |
| Other disorders of middle ear and mastoid | 1.2(0.1-9.9) | 0.898130619 | 1 | 8780 | 0.930857659 |
| Otosclerosis | 3.8(0.6-22.8) | 0.142558653 | 2 | 8770 | 0.272400806 |
| Disorders of vestibular function | 1(0.7-1.5) | 0.94907314 | 27 | 8530 | 0.966346489 |
| Conductive and sensorineural hearing loss | 1.6(1.2-2.2) | 0.00242071 | 53 | 8240 | 0.010593862 |
| Other hearing loss | 1.3(0.8-2) | 0.332167817 | 21 | 8650 | 0.500712228 |
| Otalgia and effusion of ear | 1.7(0.6-4.7) | 0.283952457 | 5 | 8760 | 0.444494808 |
| Other disorders of ear, not elsewhere classified | 1.2(0.7-1.9) | 0.482389908 | 20 | 8590 | 0.645831226 |
| Postprocedural disorders of ear and mastoid process, not elsewhere | 1.2(0.1-10.4) | 0.86522813 | 1 | 8750 | 0.905264393 |
| Rheumatic tricuspid valve diseases | 2.6(0.8-8.6) | 0.11645394 | 4 | 8780 | 0.236983769 |
| Essential (primary) hypertension | 1.3(1.1-1.5) | 0.001232736 | 209 | 6640 | 0.006118581 |
| Hypertensive heart disease | 1.8(0.9-3.6) | 0.076126138 | 11 | 8690 | 0.164804991 |
| Hypertensive renal disease | 5.8(2.5-13.3) | 3.38951E-05 | 11 | 8750 | 0.000265295 |
| Secondary hypertension | 2.5(1-6.5) | 0.056629382 | 6 | 8680 | 0.129878063 |
| Angina pectoris | 1.5(1.1-2) | 0.003874003 | 58 | 8080 | 0.015216587 |
| Acute myocardial infarction | 2(1.5-2.6) | 6.36087E-07 | 70 | 8350 | 8.35121E-06 |
| Chronic ischaemic heart disease | 1.5(1.2-1.9) | 0.000739105 | 81 | 8090 | 0.003856611 |
| Pulmonary embolism | 2.1(1.3-3.4) | 0.003344124 | 22 | 8690 | 0.013888353 |
| Other pulmonary heart diseases | 3.6(1.8-7.2) | 0.00024642 | 13 | 8740 | 0.001519592 |
| Other diseases of pulmonary vessels | 3.2(0.3-29.1) | 0.309206122 | 1 | 8790 | 0.476169201 |
| Acute pericarditis | 2.3(0.7-7.4) | 0.158813494 | 4 | 8760 | 0.294383498 |
| Other diseases of pericardium | 5.3(2.4-11.7) | 3.00389E-05 | 12 | 8740 | 0.000239722 |
| Acute and subacute endocarditis | 2.4(0.9-6.9) | 0.089729258 | 5 | 8760 | 0.18632555 |
| Nonrheumatic mitral valve disorders | 2.2(1.2-4) | 0.009253379 | 15 | 8680 | 0.030401513 |
| Nonrheumatic aortic valve disorders | 2.7(1.9-3.8) | 9.35203E-09 | 48 | 8570 | 1.46395E-07 |
| Nonrheumatic tricuspid valve disorders | 4.7(1.2-17.8) | 0.02208714 | 4 | 8770 | 0.060739635 |
| Endocarditis, valve unspecified | 2.9(0.3-30.6) | 0.380898232 | 1 | 8790 | 0.551692457 |
| Acute myocarditis | 8.4(1.4-51) | 0.020331615 | 3 | 8780 | 0.05706874 |
| Cardiomyopathy | 3.4(1.7-6.5) | 0.000339552 | 14 | 8710 | 0.002002864 |
| Atrioventricular and left bundle-branch block | 1.1(0.6-2.1) | 0.824207297 | 10 | 8670 | 0.887440132 |
| Other conduction disorders | 1.9(0.8-4.6) | 0.127957509 | 7 | 8730 | 0.253999209 |
| Cardiac arrest | 1.5(0.8-3.1) | 0.234104081 | 10 | 8770 | 0.385750449 |
| Paroxysmal tachycardia | 1.8(1-3.1) | 0.037145384 | 17 | 8650 | 0.093322046 |
| Atrial fibrillation and flutter | 1.6(1.3-2) | 2.28085E-05 | 106 | 8050 | 0.00018945 |
| Other cardiac arrhythmias | 1.1(0.7-1.7) | 0.630661174 | 24 | 8540 | 0.744323325 |
| Heart failure | 2.6(2.2-3.2) | 8.13493E-23 | 145 | 8240 | 5.5182E-21 |
| Complications and ill-defined descriptions of heart disease | 1.6(0.6-3.9) | 0.310036458 | 6 | 8750 | 0.476169201 |
| Subarachnoid haemorrhage | 0.8(0.2-3.4) | 0.730194723 | 2 | 8780 | 0.827825216 |
| Intracerebral haemorrhage | 0.7(0.3-1.7) | 0.402737692 | 5 | 8760 | 0.570552097 |
| Other nontraumatic intracranial haemorrhage | 3(1.5-5.8) | 0.001331651 | 13 | 8750 | 0.006452166 |
| Cerebral infarction | 1.3(1-1.8) | 0.063317973 | 50 | 8500 | 0.140056603 |
| Stroke, not specified as haemorrhage or infarction | 0.9(0.3-2.6) | 0.840921635 | 4 | 8770 | 0.891036875 |
| Occlusion and stenosis of precerebral arteries, not resulting in cerebral | 2.1(1.1-4.2) | 0.033742226 | 11 | 8710 | 0.087471884 |
| Occlusion and stenosis of cerebral arteries, not resulting in cerebral | 3(0.3-30.9) | 0.353875859 | 1 | 8790 | 0.529512775 |
| Other cerebrovascular diseases | 1.9(0.9-4) | 0.097593979 | 9 | 8730 | 0.199601756 |
| Sequelae of cerebrovascular disease | 1(0.7-1.5) | 0.823534665 | 31 | 8570 | 0.887440132 |
| Atherosclerosis | 3.7(2.6-5.4) | 1.9303E-12 | 46 | 8560 | 4.36463E-11 |
| Aortic aneurysm and dissection | 0.8(0.4-1.6) | 0.543357025 | 9 | 8690 | 0.690421808 |
| Other aneurysm and dissection | 0.8(0.2-3.6) | 0.784718136 | 2 | 8780 | 0.871882274 |
| Other peripheral vascular diseases | 3.6(2.3-5.5) | 7.71598E-09 | 33 | 8640 | 1.25616E-07 |
| Arterial embolism and thrombosis | 1.3(0.5-3) | 0.61770046 | 6 | 8750 | 0.732956522 |
| Other disorders of arteries and arterioles | 1.3(0.3-5.9) | 0.739274003 | 2 | 8770 | 0.835790331 |
| Diseases of capillaries | 1.1(0.5-2.7) | 0.795894734 | 6 | 8740 | 0.873633 |
| Phlebitis and thrombophlebitis | 1.8(1.2-2.7) | 0.002246133 | 33 | 8540 | 0.009936699 |
| Portal vein thrombosis | 0.6(0.1-4.9) | 0.657333646 | 1 | 8780 | 0.767865576 |
| Other venous embolism and thrombosis | 2.5(1.1-5.5) | 0.020819431 | 9 | 8720 | 0.058037729 |
| Varicose veins of lower extremities | 1(0.6-1.7) | 0.928064993 | 14 | 8540 | 0.956259373 |
| Other disorders of veins | 2(1.2-3.3) | 0.005450435 | 21 | 8470 | 0.019631214 |
| Oesophageal varices | 0.5(0.1-4) | 0.520027658 | 1 | 8780 | 0.674048589 |
| Other disorders of veins | 1.8(0.8-3.9) | 0.159849516 | 8 | 8750 | 0.294383498 |
| Nonspecific lymphadenitis | 1.5(0.2-13.6) | 0.712843735 | 1 | 8780 | 0.810411732 |
| Other noninfective disorders of lymphatic vessels and lymph nodes | 2.3(0.4-12) | 0.324099027 | 2 | 8780 | 0.492195164 |
| Hypotension | 1.1(0.7-1.8) | 0.685668841 | 19 | 8680 | 0.791412362 |
| Postprocedural disorders of circulatory system, not elsewhere classified | 2(0.4-9.9) | 0.408062531 | 2 | 8780 | 0.570726632 |
| Other and unspecified disorders of circulatory system | 1.6(0.2-14.8) | 0.686409248 | 1 | 8780 | 0.791412362 |
| Acute nasopharyngitis [common cold] | 1.7(0.3-8) | 0.525896897 | 2 | 8750 | 0.67734189 |
| Acute sinusitis | 2(1.1-3.7) | 0.033569029 | 13 | 8630 | 0.087471884 |
| Acute pharyngitis | 2.8(1-8.3) | 0.056096996 | 5 | 8730 | 0.129724303 |
| Acute tonsillitis | 2.2(0.9-5.3) | 0.070621496 | 7 | 8700 | 0.15370561 |
| Acute laryngitis and tracheitis | 0.7(0.1-5.2) | 0.692185176 | 1 | 8760 | 0.79581742 |
| Acute upper respiratory infections of multiple and unspecified sites | 4.1(2.9-5.7) | 3.27917E-16 | 57 | 8380 | 1.48291E-14 |
| Influenza due to identified zoonotic or pandemic influenza virus | 14.3(4.5-45.7) | 6.93536E-06 | 10 | 8750 | 7.05673E-05 |
| influenza due to identified seasonal influenza virus | 1.9(1-3.8) | 0.057607736 | 11 | 8710 | 0.130985187 |
| Influenza, virus not identified | 3.8(1.4-10.6) | 0.011216209 | 6 | 8750 | 0.035944858 |
| Viral pneumonia, not elsewhere classified | 2.3(0.4-11.6) | 0.323987946 | 2 | 8780 | 0.492195164 |
| Pneumonia due to Streptococcus pneumoniae | 6.3(2.8-14.3) | 1.03445E-05 | 12 | 8740 | 9.56865E-05 |
| Pneumonia due to Haemophilus influenzae | 3.1(1.3-7.8) | 0.014214914 | 7 | 8760 | 0.042403179 |
| Bacterial pneumonia, not elsewhere classified | 2.6(1.9-3.4) | 2.13426E-11 | 72 | 8480 | 4.34322E-10 |
| Pneumonia in diseases classified elsewhere | 2.9(0.3-31.7) | 0.390163818 | 1 | 8790 | 0.556467953 |
| Pneumonia, organism unspecified | 2.8(2.3-3.5) | 6.19329E-25 | 142 | 8190 | 5.04134E-23 |
| Acute bronchitis | 3(1.9-4.7) | 1.6728E-06 | 29 | 8590 | 1.84009E-05 |
| Acute bronchiolitis | 2.9(0.3-30.3) | 0.367926226 | 1 | 8790 | 0.542557877 |
| Unspecified acute lower respiratory infection | 3.6(1.5-8.7) | 0.004453134 | 8 | 8760 | 0.017098355 |
| Vasomotor and allergic rhinitis | 1.4(0.8-2.3) | 0.232355779 | 17 | 8620 | 0.384426025 |
| Chronic rhinitis, nasopharyngitis and pharyngitis | 0.7(0.3-1.7) | 0.405045158 | 5 | 8750 | 0.570552097 |
| Chronic sinusitis | 1.1(0.5-2.6) | 0.843621724 | 6 | 8730 | 0.891036875 |
| Nasal polyp | 1.9(0.9-4.2) | 0.117095535 | 8 | 8700 | 0.237103895 |
| Other disorders of nose and nasal sinuses | 0.8(0.4-1.6) | 0.466388496 | 8 | 8680 | 0.631275635 |
| Chronic diseases of tonsils and adenoids | 1.7(0.5-6.3) | 0.405378873 | 3 | 8760 | 0.570552097 |
| Peritonsillar abscess | 1.4(0.4-5) | 0.580607501 | 3 | 8770 | 0.706138912 |
| Chronic laryngitis and laryngotracheitis | 1.5(0.4-5.5) | 0.505151865 | 3 | 8770 | 0.667522108 |
| Diseases of vocal cords and larynx, not elsewhere classified | 2.2(1.2-4.4) | 0.017689595 | 12 | 8660 | 0.05034731 |
| Other diseases of upper respiratory tract | 1.1(0.3-3.9) | 0.839026678 | 3 | 8750 | 0.891036875 |
| Bronchitis, not specified as acute or chronic | 4(1.5-10.6) | 0.004643717 | 7 | 8750 | 0.017499935 |
| Unspecified chronic bronchitis | 2.5(0.6-9.6) | 0.189511512 | 3 | 8750 | 0.336524372 |
| Emphysema | 1.7(0.6-4.6) | 0.287613719 | 5 | 8760 | 0.448501087 |
| Other chronic obstructive pulmonary disease | 1.6(1.2-2.2) | 0.003031271 | 50 | 8510 | 0.012986604 |
| Asthma | 1.1(0.7-1.7) | 0.658440015 | 25 | 8390 | 0.767865576 |
| Bronchiectasis | 0.5(0.1-3.7) | 0.473937012 | 1 | 8770 | 0.636608461 |
| Pneumonitis due to solids and liquids | 1.1(0.5-2.7) | 0.7659619 | 6 | 8780 | 0.858805766 |
| Respiratory conditions due to other external agents | 17.3(1.9-158.9) | 0.011869274 | 3 | 8770 | 0.037740583 |
| Pulmonary oedema | 2.5(1.2-5.2) | 0.013297385 | 10 | 8770 | 0.041152596 |
| Other interstitial pulmonary diseases | 1.8(0.8-4.1) | 0.183614618 | 7 | 8740 | 0.327768199 |
| Abscess of lung and mediastinum | 5.8(1.2-28.8) | 0.030686574 | 3 | 8780 | 0.081630297 |
| Pyothorax | 2.3(0.7-7.3) | 0.171659464 | 4 | 8790 | 0.310512897 |
| Pleural effusion, not elsewhere classified | 8.2(6.2-10.8) | 1.06177E-49 | 117 | 8400 | 1.08035E-47 |
| Pleural effusion in conditions classified elsewhere | 6(1.7-20.7) | 0.004804441 | 5 | 8770 | 0.017939517 |
| Pneumothorax | 3.2(1.5-7) | 0.002865828 | 10 | 8760 | 0.012408427 |
| Other pleural conditions | 3.9(1.1-13.8) | 0.035272433 | 4 | 8780 | 0.090860001 |
| Postprocedural respiratory disorders, not elsewhere classified | 2.6(0.9-7.5) | 0.080881298 | 5 | 8770 | 0.171451501 |
| Respiratory failure, not elsewhere classified | 2.4(1.6-3.7) | 5.69387E-05 | 29 | 8720 | 0.000406562 |
| Other respiratory disorders | 1.4(0.5-4.3) | 0.518847992 | 4 | 8740 | 0.674048589 |
| Embedded and impacted teeth | 1.2(0.3-5.3) | 0.834608119 | 2 | 8770 | 0.891036875 |
| Dental caries | 3.5(1.3-9.6) | 0.01523126 | 6 | 8760 | 0.044279449 |
| Diseases of pulp and periapical tissues | 3.1(1.4-6.8) | 0.003795403 | 10 | 8710 | 0.015216587 |
| Gingivitis and periodontal diseases | 2.1(0.7-6.5) | 0.219825956 | 4 | 8740 | 0.36970729 |
| Dentofacial anomalies [including malocclusion] | 1.7(0.7-3.9) | 0.227201496 | 7 | 8740 | 0.378979545 |
| Other disorders of teeth and supporting structures | 1.7(0.5-5.1) | 0.374649506 | 4 | 8740 | 0.550477794 |
| Cysts of oral region, not elsewhere classified | 2.9(0.5-15.9) | 0.219254322 | 2 | 8780 | 0.36970729 |
| Other diseases of jaws | 17.2(3.4-86.1) | 0.000541908 | 6 | 8760 | 0.002940757 |
| Diseases of salivary glands | 2.2(1.1-4.6) | 0.031252045 | 10 | 8700 | 0.082061821 |
| Stomatitis and related lesions | 4.7(2.2-9.8) | 4.36218E-05 | 13 | 8730 | 0.000317037 |
| Other diseases of lip and oral mucosa | 2.4(1.1-5) | 0.021104336 | 10 | 8750 | 0.058431732 |
| Diseases of tongue | 1.5(0.5-4.5) | 0.473821196 | 4 | 8760 | 0.636608461 |
| Oesophagitis | 3.6(2.2-5.7) | 7.05666E-08 | 29 | 8580 | 1.02574E-06 |
| Gastro-oesophageal reflux disease | 1.8(1.3-2.6) | 0.000428241 | 44 | 8240 | 0.002375735 |
| Other diseases of oesophagus | 3(1.8-5) | 4.08887E-05 | 21 | 8600 | 0.000313994 |
| Gastric ulcer | 3(1.9-4.7) | 2.10781E-06 | 28 | 8540 | 2.19969E-05 |
| Duodenal ulcer | 2.8(1.5-5.5) | 0.002063867 | 13 | 8680 | 0.009333263 |
| Peptic ulcer, site unspecified | 1.9(0.4-9.4) | 0.428409272 | 2 | 8770 | 0.591059572 |
| Gastrojejunal ulcer | 16.9(1.8-162) | 0.014377491 | 3 | 8780 | 0.042403179 |
| Gastritis and duodenitis | 2.8(2.1-3.7) | 2.66104E-12 | 68 | 8210 | 5.70023E-11 |
| Functional dyspepsia | 1.9(1.1-3.1) | 0.016757731 | 19 | 8480 | 0.04803096 |
| Other diseases of stomach and duodenum | 2.3(1.3-4) | 0.005735607 | 16 | 8710 | 0.020477123 |
| Acute appendicitis | 0.7(0.3-1.7) | 0.391031535 | 5 | 8700 | 0.556467953 |
| Other appendicitis | 2.9(0.3-30.4) | 0.377407929 | 1 | 8780 | 0.550555653 |
| Unspecified appendicitis | 1.9(0.2-17.9) | 0.585937238 | 1 | 8780 | 0.709751357 |
| Inguinal hernia | 1.3(0.9-1.8) | 0.190173478 | 40 | 8280 | 0.336524372 |
| Femoral hernia | 1.6(0.4-5.6) | 0.490584901 | 3 | 8780 | 0.651148629 |
| Umbilical hernia | 0.8(0.3-2) | 0.630937462 | 5 | 8710 | 0.744323325 |
| Ventral hernia | 0.3(0.1-1.1) | 0.067311927 | 3 | 8660 | 0.147290078 |
| Diaphragmatic hernia | 3(2.3-4) | 5.00729E-14 | 71 | 8260 | 1.56767E-12 |
| Other abdominal hernia | 1.9(0.2-18.4) | 0.573226777 | 1 | 8790 | 0.706138912 |
| Crohn’s disease [regional enteritis] | 1(0.3-3.3) | 0.955052741 | 3 | 8700 | 0.966752153 |
| Ulcerative colitis | 2.6(1.2-5.8) | 0.015458679 | 9 | 8690 | 0.044621862 |
| Other noninfective gastroenteritis and colitis | 4.7(3.2-7) | 7.35214E-15 | 45 | 8470 | 2.72029E-13 |
| Vascular disorders of intestine | 3.1(1.6-5.7) | 0.000412474 | 15 | 8750 | 0.002331626 |
| Paralytic ileus and intestinal obstruction without hernia | 1.3(0.7-2.2) | 0.38383519 | 15 | 8630 | 0.553974902 |
| Diverticular disease of intestine | 1.7(1.3-2.3) | 0.000189894 | 58 | 8390 | 0.001226776 |
| Irritable bowel syndrome | 2.2(1.3-3.9) | 0.005098796 | 17 | 8610 | 0.01852866 |
| Other functional intestinal disorders | 2.8(2.2-3.6) | 8.20278E-17 | 96 | 8320 | 4.17316E-15 |
| Fissure and fistula of anal and rectal regions | 4.6(2.5-8.3) | 7.70734E-07 | 19 | 8640 | 9.80278E-06 |
| Abscess of anal and rectal regions | 2.4(1-5.7) | 0.052491019 | 7 | 8710 | 0.1242084 |
| Other diseases of anus and rectum | 1.7(1.1-2.6) | 0.013559311 | 28 | 8580 | 0.041183879 |
| Other diseases of intestine | 1.9(1.2-3) | 0.004056864 | 26 | 8670 | 0.015725177 |
| Haemorrhoids and perianal venous thrombosis | 2(1.3-3) | 0.001549285 | 29 | 8680 | 0.007418343 |
| Peritonitis | 2(0.6-6) | 0.246437775 | 4 | 8760 | 0.396866857 |
| Other disorders of peritoneum | 1.2(0.1-10) | 0.888763573 | 1 | 8790 | 0.925132414 |
| Alcoholic liver disease | 3.3(1.3-8.3) | 0.012301876 | 7 | 8780 | 0.038812895 |
| Toxic liver disease | 5.8(1.4-23.1) | 0.013346788 | 4 | 8760 | 0.041152596 |
| Hepatic failure, not elsewhere classified | 2.6(1-6.8) | 0.051647316 | 6 | 8770 | 0.122926652 |
| Fibrosis and cirrhosis of liver | 1(0.2-4.7) | 0.95545038 | 2 | 8780 | 0.966752153 |
| Other inflammatory liver diseases | 0.9(0.2-4.2) | 0.94490468 | 2 | 8770 | 0.966346489 |
| Other diseases of liver | 1.7(0.9-3.5) | 0.123696979 | 10 | 8720 | 0.24746923 |
| Cholelithiasis | 1.5(1-2.2) | 0.036095188 | 33 | 8410 | 0.091382762 |
| Cholecystitis | 1.9(1-3.6) | 0.040476185 | 13 | 8730 | 0.100450045 |
| Other diseases of gallbladder | 2.9(0.9-9.7) | 0.082034718 | 4 | 8790 | 0.172103764 |
| Other diseases of biliary tract | 1.9(0.8-4.9) | 0.164605084 | 6 | 8730 | 0.300422731 |
| Acute pancreatitis | 2.4(1.3-4.4) | 0.003888268 | 15 | 8660 | 0.015216587 |
| Other diseases of pancreas | 1.5(0.4-5.1) | 0.555730904 | 3 | 8760 | 0.695946086 |
| Intestinal malabsorption | 0.8(0.2-3.7) | 0.815954306 | 2 | 8760 | 0.884622564 |
| Postprocedural disorders of digestive system, not elsewhere classified | 1.1(0.2-4.7) | 0.936168713 | 2 | 8750 | 0.962173399 |
| Other diseases of digestive system | 2.2(1.6-2.9) | 3.9409E-07 | 58 | 8390 | 5.53084E-06 |
| Pyogenic arthritis | 2.9(1.5-5.6) | 0.001924773 | 13 | 8700 | 0.008802053 |
| Reactive arthropathies | 2.2(0.8-6.2) | 0.129807949 | 5 | 8740 | 0.253999209 |
| Seropositive rheumatoid arthritis | 1.7(0.8-3.8) | 0.17296686 | 8 | 8670 | 0.311493415 |
| Other rheumatoid arthritis | 1.6(0.8-3.4) | 0.21051423 | 9 | 8670 | 0.361198273 |
| Psoriatic and enteropathic arthropathies | 1.9(0.7-5.2) | 0.218591081 | 5 | 8740 | 0.36970729 |
| Gout | 2.4(1.6-3.5) | 1.22128E-05 | 35 | 8600 | 0.000108057 |
| Other crystal arthropathies | 1.5(0.4-5.1) | 0.558727043 | 3 | 8780 | 0.69755186 |
| Other arthritis | 2.7(1.6-4.4) | 0.00010498 | 23 | 8640 | 0.000724182 |
| Polyarthrosis | 1.2(0.5-3.2) | 0.672126231 | 5 | 8710 | 0.779360046 |
| Coxarthrosis [arthrosis of hip] | 0.8(0.5-1.2) | 0.228401615 | 22 | 8490 | 0.379426357 |
| Gonarthrosis [arthrosis of knee] | 0.8(0.6-1.2) | 0.258660657 | 38 | 8300 | 0.412842695 |
| Arthrosis of first carpometacarpal joint | 2.1(1.1-4) | 0.030319512 | 12 | 8680 | 0.081184482 |
| Other arthrosis | 0.6(0.4-1) | 0.054727502 | 19 | 8540 | 0.12868692 |
| Acquired deformities of fingers and toes | 0.9(0.6-1.5) | 0.800007913 | 20 | 8540 | 0.873633 |
| Other acquired deformities of limbs | 1.5(0.8-3) | 0.202456385 | 11 | 8680 | 0.35364699 |
| Disorders of patella | 0.8(0.2-3.3) | 0.707521099 | 2 | 8740 | 0.80661369 |
| Internal derangement of knee | 0.8(0.4-1.4) | 0.366117598 | 12 | 8490 | 0.541854045 |
| Other specific joint derangements | 0.7(0.3-1.9) | 0.51153572 | 5 | 8720 | 0.671149836 |
| Other joint disorders, not elsewhere classified | 1.8(1.3-2.5) | 0.000186777 | 53 | 8360 | 0.001226103 |
| Other necrotizing vasculopathies | 1.1(0.2-4.8) | 0.949726279 | 2 | 8750 | 0.966346489 |
| Systemic lupus erythematosus | 5.5(0.4-87.2) | 0.224473077 | 1 | 8780 | 0.37596931 |
| Other systemic involvement of connective tissue | 1.8(1-3) | 0.039557232 | 17 | 8660 | 0.098771738 |
| Scoliosis | 1.5(0.4-5.3) | 0.554673383 | 3 | 8760 | 0.695946086 |
| Ankylosing spondylitis | 1.9(0.4-9.4) | 0.428108126 | 2 | 8770 | 0.591059572 |
| Other inflammatory spondylopathies | 1.7(0.7-4.1) | 0.277900122 | 6 | 8750 | 0.436700191 |
| Spondylosis | 1.9(1.1-3.5) | 0.026858626 | 15 | 8690 | 0.073365509 |
| Other spondylopathies | 1.4(1-1.9) | 0.042852452 | 46 | 8530 | 0.105065951 |
| Cervical disc disorders | 1.2(0.5-2.6) | 0.706724205 | 7 | 8720 | 0.80661369 |
| Other intervertebral disc disorders | 2.2(1.4-3.5) | 0.000300049 | 28 | 8600 | 0.001795883 |
| Other dorsopathies, not elsewhere classified | 1.6(0.7-3.7) | 0.271778239 | 7 | 8730 | 0.428735439 |
| Dorsalgia | 2.2(1.8-2.7) | 5.44007E-13 | 120 | 7900 | 1.38382E-11 |
| Myositis | 1.7(0.3-8.1) | 0.527620418 | 2 | 8780 | 0.677418013 |
| Other disorders of muscle | 1.1(0.5-2.6) | 0.742842147 | 7 | 8700 | 0.837497933 |
| Synovitis and tenosynovitis | 0.8(0.5-1.5) | 0.533737142 | 14 | 8600 | 0.683116406 |
| Spontaneous rupture of synovium and tendon | 1.1(0.4-2.9) | 0.843895385 | 5 | 8750 | 0.891036875 |
| Other disorders of synovium and tendon | 1.3(0.7-2.6) | 0.379873914 | 11 | 8610 | 0.551692457 |
| Soft tissue disorders related to use, overuse and pressure | 1.4(0.9-2.3) | 0.128958814 | 21 | 8640 | 0.253999209 |
| Other bursopathies | 0.4(0.1-1.9) | 0.267559479 | 2 | 8770 | 0.423874069 |
| Fibroblastic disorders | 0.5(0.2-1.3) | 0.129667043 | 4 | 8690 | 0.253999209 |
| Shoulder lesions | 1.1(0.7-1.5) | 0.777244132 | 32 | 8530 | 0.868010628 |
| Enthesopathies of lower limb, excluding foot | 0.9(0.3-2.3) | 0.799750083 | 5 | 8720 | 0.873633 |
| Other enthesopathies | 1.9(1.1-3.2) | 0.0183527 | 18 | 8610 | 0.051871866 |
| Other soft tissue disorders, not elsewhere classified | 1.5(1.2-1.8) | 1.63805E-05 | 142 | 7560 | 0.000138893 |
| Osteoporosis with pathological fracture | 1.6(0.9-3) | 0.139472198 | 12 | 8690 | 0.267760305 |
| Osteoporosis without pathological fracture | 1.6(1-2.5) | 0.056801708 | 22 | 8630 | 0.129878063 |
| Disorders of continuity of bone | 0.9(0.3-2.3) | 0.799874926 | 5 | 8750 | 0.873633 |
| Other disorders of bone density and structure | 0.3(0-2) | 0.207458842 | 1 | 8750 | 0.359301059 |
| Osteomyelitis | 3.1(1.4-6.9) | 0.006254287 | 9 | 8760 | 0.021943919 |
| Osteonecrosis | 1.6(0.5-4.7) | 0.428116688 | 4 | 8760 | 0.591059572 |
| Other disorders of bone | 2.4(0.7-7.6) | 0.154641559 | 4 | 8770 | 0.288711535 |
| Osteopathies in diseases classified elsewhere | 2.2(0.6-8.1) | 0.255137236 | 3 | 8780 | 0.408822264 |
| Other acquired deformities of musculoskeletal system and connective | 5.8(0.4-93.1) | 0.21121668 | 1 | 8770 | 0.361198273 |
| Postprocedural musculoskeletal disorders, not elsewhere classified | 0.9(0.2-4.1) | 0.898837985 | 2 | 8780 | 0.930857659 |
| Biomechanical lesions, not elsewhere classified | 0.7(0.2-2.9) | 0.602161153 | 2 | 8780 | 0.720822321 |
| Chronic nephritic syndrome | 1(0.1-8) | 0.97449584 | 1 | 8780 | 0.979308165 |
| Nephrotic syndrome | 1.4(0.2-12.8) | 0.749374742 | 1 | 8780 | 0.842529061 |
| Glomerular disorders in diseases classified elsewhere | 2.3(0.5-12) | 0.306181458 | 2 | 8780 | 0.473824537 |
| Acute tubulo-interstitial nephritis | 2.5(1.7-3.7) | 1.97953E-06 | 37 | 8600 | 2.12018E-05 |
| Chronic tubulo-interstitial nephritis | 2.5(0.6-9.8) | 0.193485997 | 3 | 8770 | 0.339434486 |
| Tubulo-interstitial nephritis, not specified as acute or chronic | 1.5(0.3-6.8) | 0.633702071 | 2 | 8760 | 0.745424112 |
| Obstructive and reflux uropathy | 2.3(1.3-3.9) | 0.003131529 | 18 | 8720 | 0.013276379 |
| Acute renal failure | 3.1(2.2-4.4) | 1.9076E-10 | 47 | 8670 | 3.52906E-09 |
| Chronic kidney disease | 3.4(2.6-4.3) | 0 | 86 | 8580 | 0 |
| Unspecified kidney failure | 4.4(2.8-6.8) | 3.6628E-11 | 34 | 8730 | 7.09886E-10 |
| Calculus of kidney and ureter | 1.7(1.2-2.5) | 0.004892262 | 36 | 8360 | 0.018101368 |
| Calculus of lower urinary tract | 1.5(0.6-4.1) | 0.386225356 | 5 | 8740 | 0.554165028 |
| Disorders resulting from impaired renal tubular function | 7.1(1.9-26.4) | 0.003308039 | 5 | 8780 | 0.013880124 |
| Other disorders of kidney and ureter, not elsewhere classified | 3.7(1.6-8.5) | 0.00189999 | 9 | 8740 | 0.008787453 |
| Cystitis | 1.7(1.1-2.6) | 0.008131977 | 31 | 8530 | 0.028288161 |
| Neuromuscular dysfunction of bladder, not elsewhere classified | 2.9(1.5-5.3) | 0.000860253 | 15 | 8720 | 0.004376538 |
| Other disorders of bladder | 4.1(1.9-8.5) | 0.000193349 | 12 | 8730 | 0.001229578 |
| Urethritis and urethral syndrome | 0.8(0.2-3.7) | 0.817243449 | 2 | 8740 | 0.884622564 |
| Other disorders of urinary system | 1.4(1.2-1.8) | 0.000839987 | 106 | 8000 | 0.004327526 |
| Hyperplasia of prostate | 1.2(0.8-1.6) | 0.362415367 | 42 | 8280 | 0.540304228 |
| Inflammatory diseases of prostate | 2.5(1.2-4.8) | 0.009262378 | 12 | 8700 | 0.030401513 |
| Hydrocele and spermatocele | 2.5(1.2-5.1) | 0.010984008 | 11 | 8680 | 0.035480088 |
| Orchitis and epididymitis | 3.3(1.7-6.5) | 0.000592864 | 13 | 8680 | 0.003174943 |
| Male infertility | 2.7(1.3-5.8) | 0.008410432 | 10 | 8670 | 0.02876509 |
| Inflammatory disorders of male genital organs, not elsewhere classified | 11.3(1-124.8) | 0.047710177 | 2 | 8780 | 0.114899657 |
| Inflammatory disorders of breast | 2.1(0.2-21.3) | 0.545487646 | 1 | 8760 | 0.690421808 |
| Hypertrophy of breast | 1.1(0.3-3.9) | 0.845717178 | 3 | 8740 | 0.891036875 |
| Unspecified lump in breast | 1.1(0.4-3.1) | 0.922606682 | 4 | 8700 | 0.95304802 |
| Other disorders of breast | 1.6(0.7-3.6) | 0.29088869 | 7 | 8710 | 0.451876706 |
| Other inflammation of vagina and vulva | 1(0.5-1.8) | 0.979373166 | 12 | 8640 | 0.981785415 |
| Female genital prolapse | 0.4(0.2-0.9) | 0.027999472 | 8 | 8600 | 0.075893613 |
| Noninflammatory disorders of ovary, fallopian tube and broad ligament | 1.6(1-2.8) | 0.060823017 | 18 | 8530 | 0.136226751 |
| Dysplasia of cervix uteri | 1.3(0.7-2.4) | 0.448005092 | 12 | 8690 | 0.613932904 |
| Other noninflammatory disorders of vulva and perineum | 2.2(1-4.7) | 0.047271992 | 9 | 8710 | 0.114522029 |
| Excessive, frequent and irregular menstruation | 2.4(1.6-3.6) | 4.34027E-05 | 31 | 8430 | 0.000317037 |
| Other abnormal uterine and vaginal bleeding | 0.5(0.2-1.5) | 0.241227185 | 4 | 8720 | 0.394295038 |
| Menopausal and other perimenopausal disorders | 1.3(1-1.7) | 0.065526287 | 59 | 8170 | 0.144157831 |
| Female infertility | 1.8(0.8-4) | 0.138398511 | 8 | 8680 | 0.266958266 |
| Postprocedural disorders of genitourinary system, not elsewhere classified | 1.5(0.5-4.6) | 0.44126379 | 4 | 8750 | 0.606737712 |

**Supplement 3 – Table 3. Results from delayed-entry analysis presented in tabular format.**

| **Category** | **Incidence rate ratio (95% CI)** | **Pvalue** | **Events** | **Person-years** | **Bonferroni** |
| --- | --- | --- | --- | --- | --- |
| Other bacterial intestinal infections | 3.9(2.3-6.8) | 1.0123E-06 | 23 | 8690 | 0.000142734 |
| Viral and other specified intestinal infections | 3.1(1.7-5.7) | 0.000215404 | 17 | 8730 | 0.030371895 |
| Other gastroenteritis and colitis of infectious and unspecified origin | 3.6(2.6-5.2) | 8.10995E-13 | 53 | 8430 | 1.1435E-10 |
| Streptococcal sepsis | 4.6(2.3-9.2) | 1.54008E-05 | 15 | 8760 | 0.002171515 |
| Other sepsis | 3(2.2-4.1) | 1.54542E-12 | 76 | 8560 | 2.17904E-10 |
| Erysipelas | 2.5(1.7-3.5) | 4.17896E-07 | 47 | 8540 | 5.89234E-05 |
| Bacterial infection of unspecified site | 2.5(1.6-4) | 0.000102807 | 28 | 8720 | 0.014495724 |
| Herpesviral [herpes simplex] infections | 2.7(1.3-5.7) | 0.009530243 | 11 | 8710 | 1 |
| Zoster [herpes zoster] | 2.8(1.6-4.7) | 0.000272943 | 21 | 8670 | 0.038484998 |
| Viral warts | 2.5(1.3-5.2) | 0.009658872 | 11 | 8700 | 1 |
| Cytomegaloviral disease | 7.2(1.9-26.8) | 0.003143235 | 7 | 8760 | 0.443196133 |
| Viral infection of unspecified site | 4.2(2.8-6.4) | 9.87055E-12 | 50 | 8490 | 1.39175E-09 |
| Candidiasis | 2.1(1.4-3.1) | 0.000256112 | 42 | 8590 | 0.036111781 |
| Streptococcus and Staphylococcus as the cause of diseases classified | 3.2(2.2-4.8) | 5.80128E-09 | 41 | 8640 | 8.1798E-07 |
| Other specified bacterial agents as the cause of diseases classified | 2.1(1.5-3) | 1.51031E-05 | 47 | 8620 | 0.002129542 |
| Viral agents as the cause of diseases classified to other chapters | 9.7(2.4-39.8) | 0.001640472 | 5 | 8780 | 0.231306564 |
| Other and unspecified infectious diseases | 3(2.1-4.3) | 6.7174E-09 | 53 | 8530 | 9.47153E-07 |
| Other nontoxic goitre | 2.4(1.2-4.6) | 0.011868793 | 14 | 8650 | 1 |
| Thyrotoxicosis [hyperthyroidism] | 3.7(2.2-6.4) | 2.47203E-06 | 22 | 8640 | 0.000348556 |
| Type 1 diabetes mellitus | 1.7(1-2.9) | 0.066841235 | 20 | 8510 | 1 |
| Type 2 diabetes mellitus | 1.3(1-1.7) | 0.031625607 | 75 | 8030 | 1 |
| Other specified diabetes mellitus | 7.7(1.7-34.6) | 0.008231207 | 4 | 8760 | 1 |
| Hyperfunction of pituitary gland | 5.8(1.5-23) | 0.012375127 | 4 | 8760 | 1 |
| Other disorders of adrenal gland | 4.3(1-19.5) | 0.054750671 | 5 | 8770 | 1 |
| Volume depletion | 3(2.1-4.4) | 1.21091E-08 | 41 | 8680 | 1.70738E-06 |
| Other disorders of fluid, electrolyte and acid–base balance | 2.3(1.6-3.1) | 7.31497E-07 | 59 | 8560 | 0.000103141 |
| Postprocedural endocrine and metabolic disorders, not elsewhere classified | 4.1(1.7-9.9) | 0.001306309 | 9 | 8730 | 0.184189526 |
| Dementia in Alzheimer disease (G30.-†) | 1.4(1.1-1.8) | 0.006104015 | 102 | 8160 | 0.860666122 |
| Meningitis due to other and unspecified causes | 11.6(1.1-127.7) | 0.044457082 | 3 | 8770 | 1 |
| Encephalitis, myelitis and encephalomyelitis | 11.9(1.1-125.7) | 0.039760028 | 3 | 8780 | 1 |
| Other extrapyramidal and movement disorders | 2.2(1-4.5) | 0.038401297 | 12 | 8660 | 1 |
| Migraine | 2.3(1.3-4) | 0.003874817 | 17 | 8630 | 0.546349177 |
| Other polyneuropathies | 3.8(2.1-7) | 1.5313E-05 | 18 | 8680 | 0.002159139 |
| Other inflammation of eyelid | 1.5(1-2.3) | 0.080565845 | 31 | 8560 | 1 |
| Other disorders of eyelid | 1.8(1.2-2.7) | 0.002046403 | 39 | 8500 | 0.288542778 |
| Disorders of lacrimal system | 2.5(1.9-3.3) | 1.31367E-10 | 80 | 8150 | 1.85228E-08 |
| Disorders of orbit | 9.7(2.3-41.5) | 0.002253817 | 6 | 8740 | 0.31778826 |
| Conjunctivitis | 3.6(2.6-5.1) | 1.17399E-14 | 60 | 8280 | 1.65532E-12 |
| Other disorders of conjunctiva | 7.4(4.8-11.3) | 0 | 54 | 8450 | 0 |
| Keratitis | 1.9(1.2-3.1) | 0.008330084 | 26 | 8570 | 1 |
| Other disorders of cornea | 2(1.1-3.4) | 0.014235688 | 19 | 8700 | 1 |
| Iridocyclitis | 2.2(1.3-3.6) | 0.003059222 | 22 | 8630 | 0.431350363 |
| Other disorders of iris and ciliary body | 15.7(4.2-58.3) | 3.80278E-05 | 8 | 8760 | 0.005361926 |
| Senile cataract | 1.3(1.1-1.5) | 0.001926713 | 177 | 7530 | 0.271666576 |
| Retinal vascular occlusions | 1.4(0.7-2.8) | 0.271449588 | 18 | 8670 | 1 |
| Other retinal disorders | 1.5(1.2-2) | 0.001256663 | 122 | 7840 | 0.177189475 |
| Retinal disorders in diseases classified elsewhere | 1.7(0.9-3) | 0.076718311 | 23 | 8510 | 1 |
| Disorders of vitreous body | 1.7(1.3-2.2) | 0.000211319 | 78 | 8120 | 0.02979593 |
| Visual disturbances | 1.8(1.3-2.6) | 0.000891249 | 42 | 8540 | 0.125666129 |
| Visual impairment including blindness (binocular or monocular) | 2.6(1.4-4.8) | 0.003097792 | 14 | 8730 | 0.436788698 |
| Otitis externa | 3(1.9-4.7) | 8.47016E-07 | 32 | 8550 | 0.000119429 |
| Other disorders of external ear | 2.1(1.5-3.1) | 2.6006E-05 | 43 | 8480 | 0.003666851 |
| Suppurative and unspecified otitis media | 3.7(1.8-7.6) | 0.000408928 | 14 | 8670 | 0.057658831 |
| Conductive and sensorineural hearing loss | 1.5(1.1-2.1) | 0.012746517 | 53 | 8240 | 1 |
| Essential (primary) hypertension | 1.1(1-1.3) | 0.096156733 | 209 | 6640 | 1 |
| Hypertensive renal disease | 5.9(2.6-13.5) | 2.96323E-05 | 11 | 8750 | 0.004178151 |
| Angina pectoris | 1.4(1.1-2) | 0.022719728 | 58 | 8080 | 1 |
| Acute myocardial infarction | 2(1.5-2.7) | 1.03788E-06 | 70 | 8350 | 0.000146341 |
| Chronic ischaemic heart disease | 1.5(1.2-2) | 0.001404906 | 81 | 8090 | 0.198091756 |
| Pulmonary embolism | 2(1.2-3.2) | 0.009128873 | 22 | 8690 | 1 |
| Other pulmonary heart diseases | 3.4(1.7-6.8) | 0.00074677 | 13 | 8740 | 0.105294581 |
| Other diseases of pericardium | 5.3(2.4-12.1) | 6.07717E-05 | 12 | 8740 | 0.008568813 |
| Nonrheumatic mitral valve disorders | 2.1(1.1-3.8) | 0.018758431 | 15 | 8680 | 1 |
| Nonrheumatic aortic valve disorders | 2.7(1.9-3.8) | 4.13184E-08 | 48 | 8570 | 5.82589E-06 |
| Cardiomyopathy | 3(1.5-6.1) | 0.002003583 | 14 | 8710 | 0.282505214 |
| Atrial fibrillation and flutter | 1.5(1.2-1.9) | 0.000173831 | 106 | 8050 | 0.024510163 |
| Heart failure | 2.6(2.1-3.1) | 1.16386E-19 | 145 | 8240 | 1.64105E-17 |
| Other nontraumatic intracranial haemorrhage | 2.8(1.4-5.5) | 0.003230748 | 13 | 8750 | 0.455535441 |
| Atherosclerosis | 3.8(2.6-5.5) | 4.03837E-12 | 46 | 8560 | 5.69411E-10 |
| Other peripheral vascular diseases | 3.5(2.2-5.5) | 5.12916E-08 | 33 | 8640 | 7.23212E-06 |
| Phlebitis and thrombophlebitis | 2(1.3-3) | 0.000790309 | 33 | 8540 | 0.111433633 |
| Other disorders of veins | 2.2(1.3-3.7) | 0.003725915 | 21 | 8470 | 0.525354077 |
| Acute upper respiratory infections of multiple and unspecified sites | 3.6(2.5-5.1) | 2.88991E-12 | 57 | 8380 | 4.07477E-10 |
| Influenza due to identified zoonotic or pandemic influenza virus | 14.4(4.5-46.1) | 6.54584E-06 | 10 | 8750 | 0.000922963 |
| Influenza, virus not identified | 2.6(0.8-8.3) | 0.11709795 | 6 | 8750 | 1 |
| Pneumonia due to Streptococcus pneumoniae | 5.8(2.4-14.1) | 8.46786E-05 | 12 | 8740 | 0.011939685 |
| Pneumonia due to Haemophilus influenzae | 3.2(1.3-7.8) | 0.013042536 | 7 | 8760 | 1 |
| Bacterial pneumonia, not elsewhere classified | 2.3(1.7-3.1) | 1.51199E-08 | 72 | 8480 | 2.13191E-06 |
| Pneumonia, organism unspecified | 2.7(2.2-3.3) | 4.74538E-20 | 142 | 8190 | 6.69098E-18 |
| Acute bronchitis | 2.3(1.4-3.8) | 0.001109445 | 29 | 8590 | 0.156431749 |
| Unspecified acute lower respiratory infection | 3.5(1.4-8.8) | 0.009154485 | 8 | 8760 | 1 |
| Bronchitis, not specified as acute or chronic | 4.4(1.5-12.6) | 0.006504437 | 7 | 8750 | 0.917125552 |
| Other chronic obstructive pulmonary disease | 1.4(1-2) | 0.038871947 | 50 | 8510 | 1 |
| Respiratory conditions due to other external agents | 17.5(1.9-159.8) | 0.011261401 | 3 | 8770 | 1 |
| Pulmonary oedema | 2.1(1-4.7) | 0.060673878 | 10 | 8770 | 1 |
| Pleural effusion, not elsewhere classified | 8.1(6.1-10.8) | 8.75308E-46 | 117 | 8400 | 1.23418E-43 |
| Pleural effusion in conditions classified elsewhere | 4.8(1.3-18.1) | 0.018930628 | 5 | 8770 | 1 |
| Pneumothorax | 3.1(1.4-7) | 0.005874261 | 10 | 8760 | 0.828270867 |
| Respiratory failure, not elsewhere classified | 1.9(1.2-3.1) | 0.006736457 | 29 | 8720 | 0.949840396 |
| Dental caries | 2.6(0.8-8.6) | 0.107782356 | 6 | 8760 | 1 |
| Diseases of pulp and periapical tissues | 3.2(1.4-7.3) | 0.005115121 | 10 | 8710 | 0.721232091 |
| Other diseases of jaws | 17.4(3.5-87.2) | 0.000523603 | 6 | 8760 | 0.073828015 |
| Stomatitis and related lesions | 4(1.8-8.7) | 0.00047068 | 13 | 8730 | 0.066365812 |
| Oesophagitis | 3.1(1.9-5.1) | 6.2433E-06 | 29 | 8580 | 0.000880305 |
| Gastro-oesophageal reflux disease | 1.7(1.2-2.5) | 0.002217122 | 44 | 8240 | 0.312614248 |
| Other diseases of oesophagus | 2.2(1.2-4) | 0.008053379 | 21 | 8600 | 1 |
| Gastric ulcer | 2.4(1.4-4) | 0.000676168 | 28 | 8540 | 0.095339656 |
| Duodenal ulcer | 1.6(0.7-3.7) | 0.262220345 | 13 | 8680 | 1 |
| Gastritis and duodenitis | 2.7(2-3.7) | 2.32019E-10 | 68 | 8210 | 3.27147E-08 |
| Functional dyspepsia | 1.9(1.1-3.3) | 0.01506345 | 19 | 8480 | 1 |
| Other diseases of stomach and duodenum | 2.2(1.2-4.1) | 0.007644905 | 16 | 8710 | 1 |
| Diaphragmatic hernia | 2.9(2.1-3.8) | 4.7172E-12 | 71 | 8260 | 6.65125E-10 |
| Ulcerative colitis | 2.5(1.1-5.7) | 0.029636346 | 9 | 8690 | 1 |
| Other noninfective gastroenteritis and colitis | 4.6(3.1-6.8) | 1.411E-13 | 45 | 8470 | 1.98951E-11 |
| Vascular disorders of intestine | 2.9(1.5-5.6) | 0.001664585 | 15 | 8750 | 0.234706508 |
| Diverticular disease of intestine | 1.7(1.3-2.3) | 0.000507491 | 58 | 8390 | 0.071556179 |
| Irritable bowel syndrome | 2.6(1.5-4.6) | 0.001050461 | 17 | 8610 | 0.148115056 |
| Other functional intestinal disorders | 2.7(2.1-3.5) | 1.60591E-14 | 96 | 8320 | 2.26433E-12 |
| Fissure and fistula of anal and rectal regions | 3.7(1.9-7.2) | 0.000124062 | 19 | 8640 | 0.017492689 |
| Other diseases of anus and rectum | 1.6(1-2.5) | 0.042082146 | 28 | 8580 | 1 |
| Other diseases of intestine | 2(1.2-3.1) | 0.003722717 | 26 | 8670 | 0.524903042 |
| Haemorrhoids and perianal venous thrombosis | 1.7(1.1-2.7) | 0.018800096 | 29 | 8680 | 1 |
| Alcoholic liver disease | 3.4(1.2-9.3) | 0.017376723 | 7 | 8780 | 1 |
| Toxic liver disease | 7.8(1.7-34.7) | 0.007251468 | 4 | 8760 | 1 |
| Acute pancreatitis | 2.3(1.2-4.4) | 0.010011321 | 15 | 8660 | 1 |
| Other diseases of digestive system | 2.1(1.5-2.8) | 6.28432E-06 | 58 | 8390 | 0.000886089 |
| Pyogenic arthritis | 3(1.5-5.9) | 0.001242742 | 13 | 8700 | 0.175226684 |
| Gout | 2.3(1.5-3.5) | 0.000113902 | 35 | 8600 | 0.016060216 |
| Other arthritis | 2.6(1.6-4.3) | 0.000180178 | 23 | 8640 | 0.025405063 |
| Other joint disorders, not elsewhere classified | 1.8(1.3-2.5) | 0.000289572 | 53 | 8360 | 0.040829638 |
| Other intervertebral disc disorders | 2.5(1.6-3.9) | 9.45036E-05 | 28 | 8600 | 0.013325015 |
| Dorsalgia | 2.1(1.6-2.6) | 1.39072E-10 | 120 | 7900 | 1.96091E-08 |
| Other soft tissue disorders, not elsewhere classified | 1.4(1.2-1.7) | 0.000536977 | 142 | 7560 | 0.075713815 |
| Osteomyelitis | 3.3(1.5-7.4) | 0.004077016 | 9 | 8760 | 0.574859226 |
| Acute tubulo-interstitial nephritis | 2.7(1.8-3.9) | 9.59003E-07 | 37 | 8600 | 0.000135219 |
| Obstructive and reflux uropathy | 2.3(1.3-4) | 0.003379248 | 18 | 8720 | 0.476474014 |
| Acute renal failure | 3.1(2.1-4.4) | 2.00163E-09 | 47 | 8670 | 2.82229E-07 |
| Chronic kidney disease | 3.2(2.4-4.1) | 0 | 86 | 8580 | 0 |
| Unspecified kidney failure | 4.3(2.8-6.8) | 1.54138E-10 | 34 | 8730 | 2.17334E-08 |
| Calculus of kidney and ureter | 1.7(1.2-2.6) | 0.004520596 | 36 | 8360 | 0.637403987 |
| Disorders resulting from impaired renal tubular function | 5.8(1.5-23.1) | 0.012896557 | 5 | 8780 | 1 |
| Other disorders of kidney and ureter, not elsewhere classified | 3.6(1.5-8.6) | 0.003863862 | 9 | 8740 | 0.544804482 |
| Cystitis | 1.6(1.1-2.5) | 0.019058126 | 31 | 8530 | 1 |
| Neuromuscular dysfunction of bladder, not elsewhere classified | 2.4(1.2-4.7) | 0.010943687 | 15 | 8720 | 1 |
| Other disorders of bladder | 3.8(1.8-8.1) | 0.000583379 | 12 | 8730 | 0.082256391 |
| Other disorders of urinary system | 1.4(1.1-1.7) | 0.006311639 | 106 | 8000 | 0.889941093 |
| Inflammatory diseases of prostate | 2.6(1.3-5.1) | 0.006569512 | 12 | 8700 | 0.92630113 |
| Hydrocele and spermatocele | 1.9(0.9-4.2) | 0.111311632 | 11 | 8680 | 1 |
| Orchitis and epididymitis | 2.8(1.4-5.8) | 0.004585607 | 13 | 8680 | 0.646570551 |
| Male infertility | 2.5(1-6.1) | 0.039787531 | 10 | 8670 | 1 |
| Excessive, frequent and irregular menstruation | 1.8(1.1-2.9) | 0.015785524 | 31 | 8430 | 1 |

**Supplements 4 – Figure summarizing all findings.**

# Figure 1 page 1
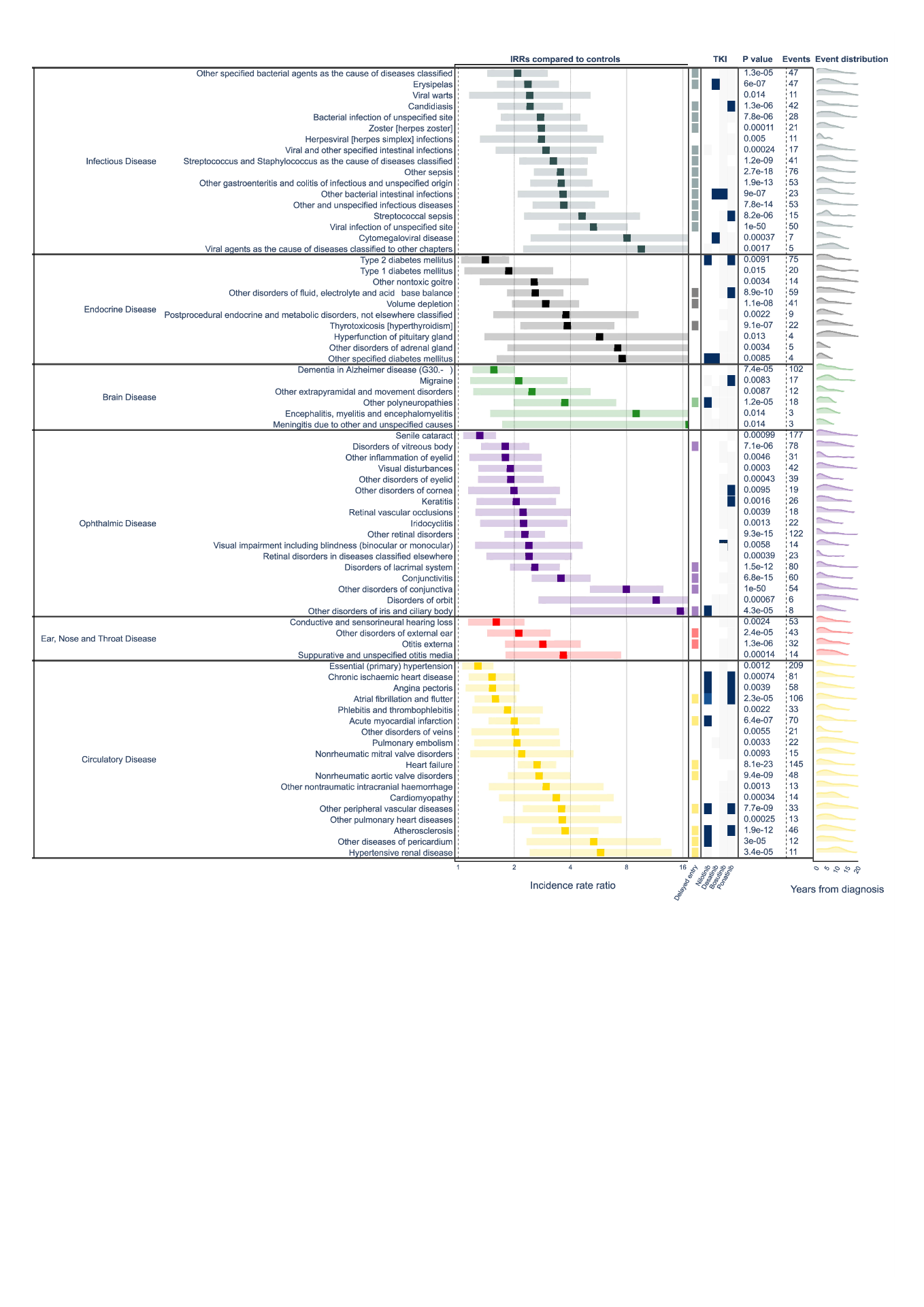


**Figure 1** page 2


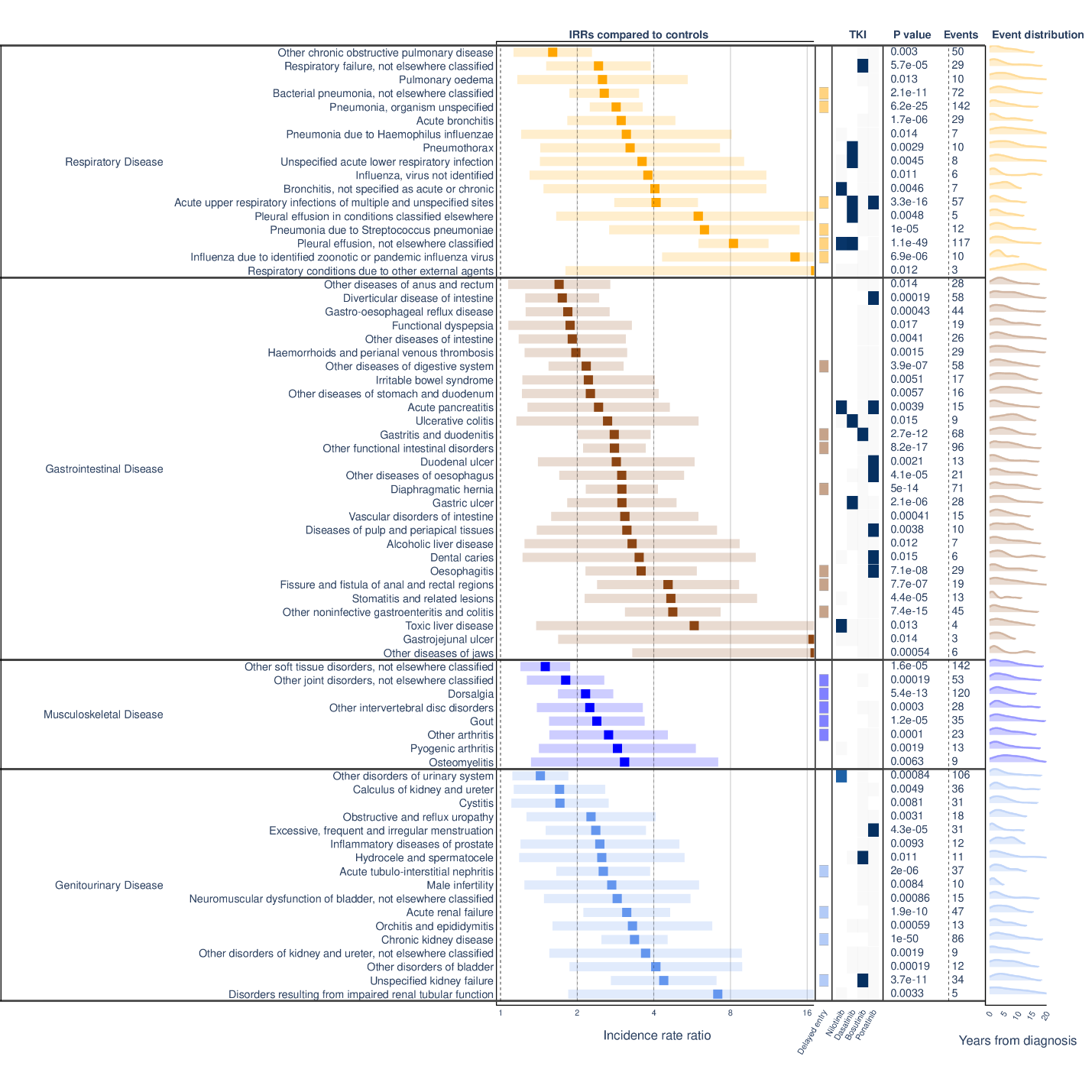


**Figure 1**. First column demonstrates incidence rate ratios (IRRs) for significant finding after FDR adjustment for the CML population as compared to the control population: strong color demonstrating the point estimate and lighter color demonstrating 95% confidence intervals. Second column demonstrates the same analysis but findings significant after Bonferroni-adjustment in a delayed entry model with 6 months from diagnosis of the significant findings from the first analysis. Third column demonstrates an un-adjusted analysis, as to multiple comparison, for significant associations between a specific TKI as compared to imatinib, in terms of the outcome. Lighter blue color demonstrates a lower IRR as compared to stronger color. Fourth and fifth column present raw p-values for the analysis in column 1 and events, respectively. Sixth column presents event distribution during follow-up from date of diagnosis.
